# Genome-Scale Reconstruction and Analysis of the *Pseudomonas putid*a KT2440 Metabolic Network Facilitates Applications in Biotechnology

**DOI:** 10.1371/journal.pcbi.1000210

**Published:** 2008-10-31

**Authors:** Jacek Puchałka, Matthew A. Oberhardt, Miguel Godinho, Agata Bielecka, Daniela Regenhardt, Kenneth N. Timmis, Jason A. Papin, Vítor A. P. Martins dos Santos

**Affiliations:** 1 Synthetic and Systems Biology Group, Helmholtz Center for Infection Research (HZI), Braunschweig, Germany; 2 Department of Biomedical Engineering, University of Virginia, Health System, Charlottesville, Virginia, United States of America; 3 Environmental Microbiology Group, Helmholtz Center for Infection Research (HZI), Braunschweig, Germany; University of Tokyo, Japan

## Abstract

A cornerstone of biotechnology is the use of microorganisms for the efficient production of chemicals and the elimination of harmful waste. *Pseudomonas putida* is an archetype of such microbes due to its metabolic versatility, stress resistance, amenability to genetic modifications, and vast potential for environmental and industrial applications. To address both the elucidation of the metabolic wiring in *P. putida* and its uses in biocatalysis, in particular for the production of non-growth-related biochemicals, we developed and present here a genome-scale constraint-based model of the metabolism of *P. putida* KT2440. Network reconstruction and flux balance analysis (FBA) enabled definition of the structure of the metabolic network, identification of knowledge gaps, and pin-pointing of essential metabolic functions, facilitating thereby the refinement of gene annotations. FBA and flux variability analysis were used to analyze the properties, potential, and limits of the model. These analyses allowed identification, under various conditions, of key features of metabolism such as growth yield, resource distribution, network robustness, and gene essentiality. The model was validated with data from continuous cell cultures, high-throughput phenotyping data, ^13^C-measurement of internal flux distributions, and specifically generated knock-out mutants. Auxotrophy was correctly predicted in 75% of the cases. These systematic analyses revealed that the metabolic network structure is the main factor determining the accuracy of predictions, whereas biomass composition has negligible influence. Finally, we drew on the model to devise metabolic engineering strategies to improve production of polyhydroxyalkanoates, a class of biotechnologically useful compounds whose synthesis is not coupled to cell survival. The solidly validated model yields valuable insights into genotype–phenotype relationships and provides a sound framework to explore this versatile bacterium and to capitalize on its vast biotechnological potential.

## Author Summary

The pseudomonads include a diverse set of bacteria whose metabolic versatility and genetic plasticity have enabled their survival in a broad range of environments. Many members of this family are able to either degrade toxic compounds or to efficiently produce high value compounds and are therefore of interest for both bioremediation and bulk chemical production. To better understand the growth and metabolism of these bacteria, we developed a large-scale mathematical model of the metabolism of *Pseudomonas putida*, a representative of the industrially relevant pseudomonads. The model was initially expanded and validated with substrate utilization data and carbon-tracking data. Next, the model was used to identify key features of metabolism such as growth yield, internal distribution of resources, and network robustness. We then used the model to predict novel strategies for the production of precursors for bioplastics of medical and industrial relevance. Such an integrated computational and experimental approach can be used to study its metabolism and to explore the potential of other industrially and environmentally important microorganisms.

## Introduction

*Pseudomonas putida* is one of the best studied species of the metabolically versatile and ubiquitous genus of the Pseudomonads [1]–[3]. As a species, it exhibits a wide biotechnological potential, with numerous strains (some of which solvent-tolerant [4],[5]) able to efficiently produce a range of bulk and fine chemicals. These features, along with their renowned stress resistance, amenability for genetic manipulation and suitability as a host for heterologous expression, make *Pseudomonas putida* particularly attractive for biocatalysis. To date, strains of *P. putida* have been employed to produce phenol, cinnamic acid, cis-cis-muconate, p-hydroxybenzoate, p-cuomarate, and myxochromide [6]–[12]. Furthermore, enzymes from *P. putida* have been employed in a variety of other biocatalytic processes, including the resolution of d/l-phenylglycinamide into d-phenylglycinamide and l-phenylglycine, production of non-proteinogenic l-amino acids, and biochemical oxidation of methylated heteroaromatic compounds for formation of heteroaromatic monocarboxylic acids [13]. However, most Pseudomonas-based applications are still in infancy largely due to a lack of knowledge of the genotype-phenotype relationships in these bacteria under conditions relevant for industrial and environmental endeavors. In an effort towards the generation of critical knowledge, the genomes of several members of the Pseudomonads have been or are currently being sequenced (http://www.genomesonline.org, http://www.pseudomonas.com), and a series of studies are underway to elucidate specific aspects of their genomic programs, physiology and behavior under various stresses (e.g., http://www.psysmo.org, http://www.probactys.org, http://www.kluyvercentre.nl).

The sequencing of *P. putida* strain KT2440, a workhorse of *P. putida* research worldwide and a microorganism Generally Recognized as Safe (GRAS certified) [1],[14], provided means to investigate the metabolic potential of the *P. putida* species, and opened avenues for the development of new biotechnological applications [2], [14]–[16]. Whole genome analysis revealed, among other features, a wealth of genetic determinants that play a role in biocatalysis, such as those for the hyper-production of polymers (such as polyhydroxyalkanoates [17], [18]) and industrially relevant enzymes, the production of epoxides, substituted catechols, enantiopure alcohols, and heterocyclic compounds 13,15. However, despite the clear breakthrough in our understanding of *P. putida* through this sequencing effort, the relationship between the genotype and the phenotype cannot be predicted simply from cataloguing and assigning gene functions to the genes found in the genome, and considerable work is still needed before the genome can be translated into a fully functioning metabolic model of value for predicting cell phenotypes [2],[14].

Constraint-based modeling is currently the only approach that enables the modeling of an organism's metabolic and transport network at genome-scale [19]. A genome-wide constraint-based model consists of a stoichiometric reconstruction of all reactions known to act in the metabolism of the organism, along with an accompanying set of constraints on the fluxes of each reaction in the system [19],[20]. A major advantage of this approach is that the model does not require knowledge on the kinetics of the reactions. These models define the organism's global metabolic space, network structural properties, and flux distribution potential, and provide a framework with which to navigate through the metabolic wiring of the cell [19]–[21].

Through various analysis techniques, constraint-based models can help predict cellular phenotypes given particular environmental conditions. Flux balance analysis (FBA) is one such technique, which relies on the optimization for an objective flux while enforcing mass balance in all modeled reactions to achieve a set of fluxes consistent with a maximal output of the objective function. When a biomass sink is chosen as the objective in FBA, the output can be correlated with growth, and the model fluxes become predictive of growth phenotypes [22],[23]. Constraint-based analysis techniques, including FBA, have been instrumental in elucidating metabolic features in a variety of organisms [20],[24],[25] and, in a few cases thus far, they have been used for concrete biotechnology endeavors [26]–[29].

However, in all previous applications in which a constraint-based approach was used to design the production of a biochemical, the studies addressed only the production of compounds that can be directly coupled to the objective function used in the underlying FBA problem. The major reason for this is that FBA-based methods predict a zero-valued flux for any reaction not directly contributing to the chosen objective. Since the production pathways of most high-added value and bulk compounds operate in parallel to growth-related metabolism, straightforward application of FBA to these biocatalytic processes fails to be a useful predictor of output. Other constraint-based analysis methods, such as Extreme Pathways and Elementary Modes analysis, are capable of analyzing non-growth related pathways in metabolism, but, due to combinatorial explosion inherent to numerical resolution of these methods, they could not be used so far to predict fluxes or phenotypes at genome-scale for guiding biocatalysis efforts [30].

To address both the elucidation of the metabolic wiring in *P. putida* and the use of *P. putida* for the production of non-growth-related biochemicals, we developed and present here a genome-scale reconstruction of the metabolic network of *Pseudomonas putida* KT2440, the subsequent analysis of its network properties through constraint-based modeling and a thorough assessment of the potential and limits of the model. The reconstruction is based on up-to-date genomic, biochemical and physiological knowledge of the bacterium. The model accounts for the function of 877 reactions that connect 886 metabolites and builds upon a constraint-based modeling framework [19],[20]. Only 6% of the reactions in the network are non gene-associated. The reconstruction process guided the refinement of the annotation of several genes. The model was validated with continuous culture experiments, substrate utilization assays (BIOLOG) [31], ^13^C-measurement of internal fluxes [32], and a specifically generated set of mutant strains. We evaluated the influence of biomass composition and maintenance values on the outcome of flux balance analysis (FBA) simulations, and utilized the metabolic reconstruction to predict internal reaction fluxes, to identify different mass-routing possibilities, and to determine necessary gene and reaction sets for growth on minimal medium. Finally, by means of a modified OptKnock approach, we utilized the model to generate hypotheses for possible improvements of the production by *P. putida* of polyhydroxyalkanoates, a class of compounds whose production consumes resources that would be otherwise used for growth. This reconstruction thus provides a modeling framework for the exploration of the metabolic capabilities of *P. putida*, which will aid in deciphering the complex genotype-phenotype relationships governing its metabolism and will help to broaden the applicability of *P. putida* strains for bioremediation and biotechnology.

## Results

### Highlights of the Model Reconstruction Process

**Figure 1:**
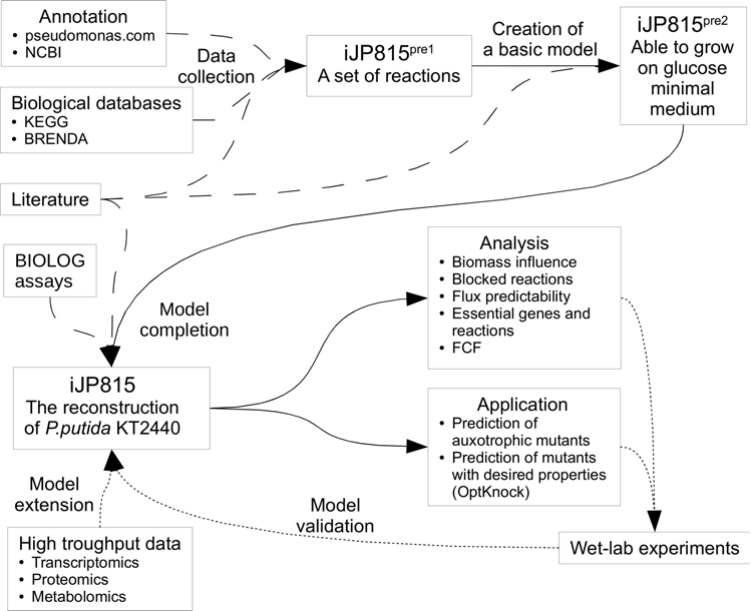
Schematic diagram of the metabolic reconstruction and analysis processes. Solid lines indicate consecutive steps of the reconstruction. Dashed lines represent information transfer. Dotted lines specify planned tasks.

We reconstructed the metabolism of *P. putida* at the genome-scale through a process summarized in Figure 1. The reconstruction process involved: (1) an initial data collection stage leading to a first pass reconstruction (iJP815^pre1^); (2) a model building stage in which simulations were performed with iJP815^pre1^ and reactions were added until the model was able to grow *in silico* on glucose minimal medium (iJP815^pre2^); and (3) a model completion stage in which BIOLOG substrate utilization data was used to guide model expansion and *in silico* viability on varied substrates. The final reconstruction, named iJP815 following an often used convention [33], consists of 824 intracellular and 62 extracellular metabolites connected by 877 reactions. Eight hundred twenty one (94%) reactions have at least one assigned gene as delineated in the gene-protein-reaction (GPR) relationships. GPR relationships are composed of Boolean logic statements that link genes to protein complexes and protein complexes to reactions via combinations of AND and OR operators. An ‘AND’ operator denotes the required presence of two or more genes for a protein to function (as in the case of multi-protein complexes), while an ‘OR’ operator denotes a redundant function that can be catalyzed by any of several genes (as in the case of isozymes). Only 56 reactions, of which nine are non-enzymatic, lack associated genes. The remaining 47 non-gene-associated, enzymatic reactions were added in order to close metabolic network gaps identified during the successive steps of the reconstruction process.

Most network gaps (27) were identified during the second round of the reconstruction and were resolved through detailed literature mining, thereby enabling iJP815 to grow *in silico* on glucose in minimal medium. The remaining gaps identified in the model completion step (Figure 1) were mostly single missing steps in the pathway for which there is experimental evidence of operation (e.g., a compound is consumed but not produced, and no alternative pathways exist). It should be noted that for some gaps, there is more than one combination of reactions with which the gap could be closed [34]. In cases where more than one gap closure method was available, the decision of which to use was made based on similarity queries to related bacteria.

**Figure 2:**
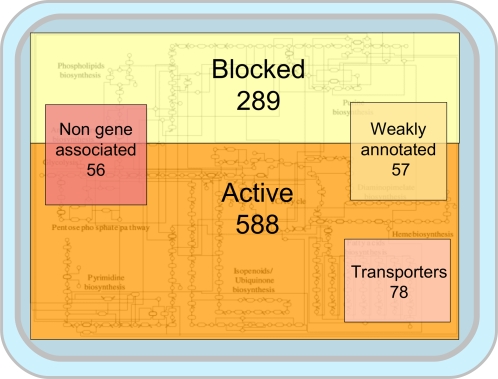
Schematic representation of various reaction classes and their interdependency. The areas of the squares correspond to the sizes of the subsets.

**Figure 3:**
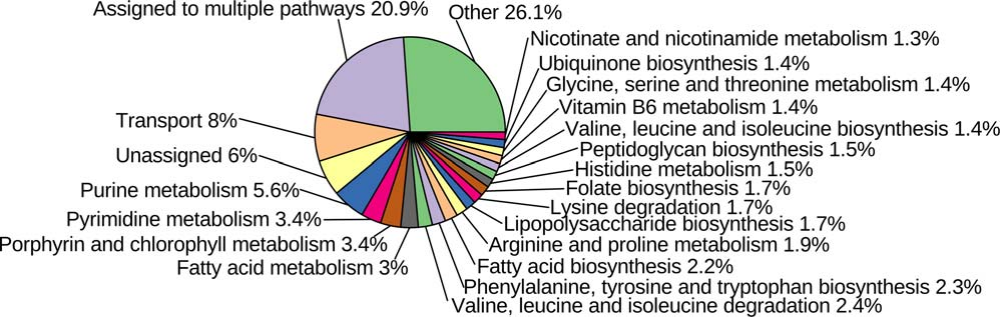
Assignment of the reactions to the particular pathways.

The iJP815 model includes 289 reactions for which non-zero flux values cannot be obtained under any environmental condition while enforcing the pseudo steady-state assumption (PSSA). We term these reactions “unconditionally blocked” meaning that they are unable to function because not all connections could be made with the information available. Three hundred sixty two metabolites that are only involved in these reactions are classified as “unbalanced metabolites”. Another important subset of model reactions is the “weakly annotated” set, which means that all the genes assigned to these 57 reactions are currently annotated as coding for “putative” or “family” proteins. The relationships between all the subsets are shown in Table 1 and Figures 2 and 3.

**Table 1:** Summary of the main characteristics of the iJP815 metabolic model.

System	Parameter	Subset			Size		
*P. putida* KT2440	Genome size				6.18 Mbp		
	Total ORFs				5446		
iJP815	Reactions	Total			877		
			Potentially active			588 (67.0%)	
			Unconditionally Blocked			289 (33.0%)	
			Well annotated			764 (87.1%)	
			Weakly annotated			57 (6.5%)	
			Non-gene-associated			56 (6.4%)	
			Transport			70 (8.0%)	
	Metabolites	Total			888		
			Internal			824 (92.8%)	
				Balanced			461 (55.9%)
				Unbalanced			363 (44.1%)
			External			64 (7.2%)	
	Genes	Total			815		
			Well annotated			701 (86.0%)	
			Weakly annotated			114 (14.0%)	

The final reconstruction accounts for the function of 815 genes, corresponding to 15% of all genes in the *P. putida* genome and to 65% (1253) of those currently assigned to the classes ‘Metabolism’ (K01100) and ‘Membrane Transport’ (K01310) in the Kyoto Encyclopedia of Genes and Genomes (KEGG) orthology classification [35]. These figures are consistent with recently published metabolic reconstructions for other prokaryotes (see Table S1).

### Model Assessment and Extension through High-Throughput Phenotyping Assays

A high-throughput BIOLOG phenotypic assay was performed on *P. putida* to validate and extend the model. In this assay, *P. putida* was tested for its ability to oxidize 95 carbon substrates in minimal medium. Of these 95 substrates, *P. putida* oxidized 45. We added 2 other carbon sources to the positive-oxidation group (l-phenylalanine and l-threonine) despite a negative BIOLOG result, since these substrates had been previously shown to be growth substrates [16] and since we confirmed these results experimentally (data not shown), giving altogether forty seven compounds utilized *in vivo*. Forty seven out of the 95 carbon sources tested were accounted for in iJP815^pre2^, enabling a comparison of these BIOLOG data with FBA simulations of iJP815 grown on *in silico* minimal medium with the respective compound as sole carbon source (see Table 2 and Table S2).

**Table 2:** Summary of the comparison with the BIOLOG substrate utilization assay.

Compounds tested	95			
Utilized compounds	47			
Reconstruction version	iJP815^pre2^		iJP815	
Tested compounds included in the model	47		51	
Utilized compounds included in the model	33		37	
Compound supply	Ext	Int	Ext	Int
True positives	14	28	23	33
True negatives	48 (14)	42 (8)	48 (14)	42 (8)
False positives	0	6	0	6
False negatives	33 (19)	20 (6)	24 (14)	14 (4)

Values in brackets indicate only those compounds that iJP815 accounts for.

The initial working version of the model (iJP815^pre2^) was able to simulate growth with 14 of the 47 BIOLOG-assayed compounds as sole carbon sources. This version of the reconstruction contained only a few transport reactions, prompting us to identify compounds that could not be utilized *in silico* simply due to the lack of a transporter. This was achieved by allowing the intracellular pool of each compound of interest to be exchanged with environment *in silico*, and by evaluating the production of biomass in each case through FBA simulations. This approach increased the number of utilizable substances to 34 but also produced six false-positives (i.e., substances that support *in silico* growth, but which gave a negative phenotype in the BIOLOG assay). These included three metabolites involved in central metabolic pathways (d-glucose 1-phosphate, d-glucose 6-phosphate and glycerol-3-phosphate), an intermediate of the l-histidine metabolism pathway (urocanate), an intermediate of branched amino acids biosynthesis (2-oxobutanoate), and the storage compound glycogen. This analysis suggests that the inability of *P. putida* to utilize these compounds *in vivo* is likely due to the lack of appropriate transport machinery.

The final *P. putida* model (iJP815) grew on 39 of the 51 compounds tested in the BIOLOG assay and that concurrently were accounted for in the model. Of these, 33 were true positives (compounds utilized in *vivo* and allowing for growth *in silico*). The mode of utilization of the remaining fourteen *in vivo* oxidized compounds (i.e., false negatives) could not be elucidated. The remaining forty two compounds posed true negatives, eight of which were accounted for in the reconstruction. Ten utilized compounds also lack transport reactions, as nothing is known about their translocation into the cell. Nevertheless, this comparison of *in silico* growth predictions with BIOLOG substrate utilization data indicates that the core metabolism of *P. putida* has been properly reconstructed.

A note of caution when comparing the BIOLOG assays with growth predictions is that this assay evaluates whether an organism is able to oxidize the tested compound and yield energy from it, which is different from growth. However, as *P. putida* is able to grow on minimal medium supplemented with these compounds, we considered the assumption to be justified.

### Model-Driven Reannotation

The reconstruction process systematizes knowledge about the metabolism of an organism, allowing the identification of errors in, and discrepancies between, various sources of data. A major value of a manual model-building effort is the careful revision of the current genome annotation, based on literature evidence encountered during the model building process, BLAST searches, and gap closures. During the reconstruction of the *P. putida* metabolic network, we discovered a number of genes that appear to have been improperly annotated in biological databases (Pseudomonas Genome Database, KEGG, NCBI). These mis-annotations arose due to a lack of information at the time of the original annotation or because knowledge that was available in the literature had been overlooked in the original annotation. In a number of other cases, the model building process has also generated new hypotheses for gene functions. For instance, our reconstruction process identified an unlikely gap in the l-lysine degradation pathway of *P. putida*. Extensive literature search and careful reannotation has provided considerable evidence that the genes PP0382 and PP5257, currently annotated as ‘carbon-hydrogen hydrolase family protein’ and ‘oxidoreductase, FAD binding’ respectively, most probably code for a ‘5-aminopentamidase’ and ‘l-pipecolate oxidase’, respectively [36]. Another example is the propanoate degradation pathway: In the iJP815^pre2^ version this pathway was complete except for one enzymatic activity, namely the 2-methylisocitrate dehydratase. Analysis of the enzymes flanking this reaction showed that all of the enzymes are encoded by genes immediately adjacent to the ORF PP2330. Inspection of this region of the genome revealed that PP2336 is annotated as “aconitate hydratase, putative”, although the flanking genes are responsible for degradation of propanoate. Analysis of PP2330 via BLAST revealed a homology of more than 99% over the whole length of the protein with the 2-methylisocitrate dehydratase from other bacteria, such as other strains of *P. putida* (GB-1, W619), *Burkholderia prymatum* STM 815, *Burkholderia multivorans* ATCC 17616, *Pseudomonas aeruginosa* PA7, and *Stenotrophomonas maltophilia* R551-3. Consequently the gene was reannotated to code for this function and the gap in propanoate degradation pathway was thus closed by addition of the corresponding GPR. In other cases, discrepancies exist between various databases, as in the case of PP5029, which is annotated in KEGG as ‘formiminoglutamase’ but in NCBI as ‘*N*-formylglutamate deformylase’. Analysis of network gaps, genomic context and sequence homology provided a strong indication that ‘*N*-formylglutamate deformylase’ is the correct annotation. In many other cases the reannotation meant changing the substrate specificity of the enzyme (which corresponds to changing the last part of the EC number). These were mainly identified by BLASTing the protein against protein sequences of other microbes and, whenever available, cross-checking the BLAST results against primary research publications. The full list of reannotations suggested by the reconstruction process is shown in Table 3.

**Table 3:** List of genes reannotated during the reconstruction process.

Gene	Old Annotation	New Annotation	Reference
PP0213	Succinate-semialdehyde dehydrogenase; EC:1.2.1.16	Glutarate-semialdehyde; dehydrogenase EC 1.2.1.20	[36]
PP0214	4-Aminobutyrate aminotransferase; EC:2.6.1.19, EC:2.6.1.22	5-Aminovalerate transaminase; EC 2.6.1.48	[36]
PP0382	Carbon-nitrogen hydrolase family protein	5-Aminopentanamidase; EC 3.5.1.30	[36]
PP0383	Tryptophan 2-monooxygenase, putative	Lysine 2-monooxygenase; EC 1.13.12.2	[36]
PP2336	Aconitate hydratase, putative; EC:4.2.1.3	2-Methylisocitrate dehydratase; EC 4.2.1.99	a
PP2432	Oxygen-insensitive NAD(P)H nitroreductase; EC:1.-.-.-	6,7-Dihydropteridine reductase; EC 1.5.1.34	a
PP3591	Malate dehydrogenase, putative; EC:1.1.1.37	Δ^1^-Piperideine-2-carboxylate reductase; EC 1.5.1.21	[36]
PP4066	Enoyl-CoA hydratase, putative; EC:4.2.1.17	Methylglutaconyl-CoA hydratase; EC 4.2.1.18	[88]
PP4065	3-Methylcrotonyl-CoA carboxylase, beta subunit, putative EC:6.4.1.3	Methylcrotonoyl-CoA carboxylase; EC 6.4.1.4	[88]
PP4067	AcCoA carboxylase, biotin carboxylase, putative; EC:6.4.1.3	Methylcrotonoyl-CoA carboxylase; EC 6.4.1.4	[88]
PP4223	Diaminobutyrate-2-oxoglutarate transaminase; EC:2.6.1.76	Putrescine aminotransferase; EC 2.6.1.82	a
PP4481	Acetylornithine aminotransferase; EC:2.6.1.11	Succinylornithine transaminase; EC 2.6.1.81	a
PP5029	Formiminoglutamase; EC:3.5.3.8	*N*-Formylglutamate deformylase; EC 3.5.1.68	a
PP5036	Atrazine chlorohydrolase	*N*-Formylglutamate deformylase; EC 3.5.1.68	a
PP5257	Oxidoreductase, FAD-binding	l-Pipecolate oxidase; EC 1.5.3.7	[36]
PP5258	Aldehyde dehydrogenase family protein; EC:1.2.1.3	l-Aminoadipate-semialdehyde dehydrogenase; EC 1.2.1.31	[36]

a Analysis of the sequence homology and genomic context information.

### Comparison of the Predicted and Measured Growth Yields and the Role of Maintenance

After completing the reconstruction, we assessed whether the model was capable of predicting the growth yield of *P. putida*, a basic property of the modeled organism. *In silico* growth yield on succinate was calculated by FBA and compared with *in vivo* growth yield measured in continuous culture [37]. If the *in silico* yield were lower than the experimental, it would indicate that the network may lack important reactions that influence the efficiency of conversion of carbon source into biomass constituents and/or energy. In fact, the calculated *in silico* yield (0.61 g_DW_⋅g_C_^−1^) was higher than the experimental yield (0.47 g_DW_⋅g_C_^−1^), indicating that some of the processes reconstructed in the network might be unrealistically efficient and/or that *P. putida* may be diverting resources into other processes not accounted for in the model. This greater efficiency of the *in silico* model versus *in vivo* growth data is also consistent with recent studies that suggest optimal growth is not necessarily the sole objective (function) of biochemical networks [38],[39].

The *in silico* growth yield is influenced not only by the structure of the metabolic network, but also by other factors including biomass composition and the growth-associated and non-growth-associated energy maintenance factors (GAM and NGAM), the values of which represent energy costs to the cell of “living” and “growing”, respectively [22]. Therefore, since both the biomass composition and the GAM/NGAM values were taken from the *E. coli* model [22],[33] due to a lack of organism-specific experimental information, we evaluated the influence of these factors on the predicted growth yield.

First, we analyzed the effects of changes in the ratios of biomass components on the iJP815 growth yield. These analyses (displayed in the Text S1, section “Assessment of the influence of the biomass composition the growth yield”) indicated that varying any single biomass constituent by 20% up or down has a less than 1% effect on the growth yield of *P. putida* (Figure S1). These results are consistent with results of a previous study on the sensitivity of growth yield to biomass composition [40]. Although it is still possible that some components of *P. putida* biomass are not present in *E. coli* or vice versa, we conclude that the use of *E. coli* biomass composition in the *P. putida* model is a justified assumption for the purpose of our application and is probably not a great contributor to the error in our predictions of growth yield.

Subsequently, the effects of changes in the GAM on the *in silico* growth yield were tested (Figure S2A and S2B). It was found that if GAM was of the same order of magnitude as the value used in the *E. coli* model (13 [mmol_ATP_⋅g_DW_^−1^), its influence is negligible, as increasing or decreasing it twofold alters the growth yield by merely 5%. A higher GAM value in *P. putida* than in *E. coli* could contribute to the discrepancy between the experimental measurements and *in silico* predictions, but it could not be the only factor unless the *E. coli* and *P. putida* values differ more than twofold, which is unlikely.

Finally, we assessed the effects of changes in the value of NGAM on *in silico* growth yield. The NGAM growth dependency is influenced by the rate of carbon source supply, and thus indirectly by the growth rate. If the carbon intake flux is low (as in the case of the experiments mentioned above, with a dilution rate of 0.05 h^−1^), the fraction of energy utilized for maintenance purposes is high and therefore so is the influence of the NGAM value on growth yield (Figure S2A). Under such low-carbon intake flux conditions, a twofold increase of the NGAM value can decrease the growth yield by about 30%. This indicates that the main cause for the discrepancy between *in vivo* and *in silico* growth yields is that the NGAM value is likely to be higher in *P. putida* than in *E. coli*. Figure S2A indicates that increasing the NGAM value from 7.6 of 12 [mmol_ATP_⋅g_DW_^−1^⋅h^−1^] would reduce the *in silico* growth yield and lead to a better match with experimental values. Consequently this NGAM value was used in subsequent FBA and Flux Variability Analysis (FVA) [41] simulations.

For a high influx of carbon source (Figure S2B) the influence of NGAM on the growth yield is low and the influence of the NGAM and GAM values on growth yield are comparable. It should be noted that, while FBA predicts the optimal growth yield, few cellular systems operate at full efficiency. Bacteria tend to “waste” or redirect energy if it is abundant [42], leading to a lower-than-optimal *in vivo* growth yield. It is also worth mentioning that maintenance values may depend on the carbon source used [43] and on environmental conditions [44]–[46].

Additionally, we computed the growth yields of *P. putida* on sole sources of three other important elements—Nitrogen (N), Phosphorous (P), and Sulfur (S)—and compared these with published experimental data from continuous cultivations [37], as shown in Table 4. Since biomass composition can play a role in the efficiency of *in silico* usage of basic elements, this analysis can aid in assessing how well the biomass equation, which is equivalent to the *E. coli* biomass reaction, reproduces the true biomass composition of *P. putida*. The yield on nitrogen differs only by 10% between *in silico* and *in vivo* experiments, which suggests that the associated metabolic network for nitrogen metabolism is well characterized in the iJP815 reconstruction. The yields on phosphorous and sulfur, however, differ by more than a factor of two between the *in vivo* and *in silico* analyses, suggesting that there may be significant differences between the biomass requirements and the metabolic networks of *P. putida* and *E. coli* for these components. The differences in yields, however, may be also caused by the change of the *in vivo* biomass composition, which decreases the fraction of compounds containing the limited element, when compared to the biomass composition while the bacterium is grown under carbon-limitation. Such changes were observed experimentally in *P. putida* for nitrogen and phosphate limitations [47]. Thus, the biomass composition of *P. putida* needs to be determined precisely in the future. However, for the purpose of this work and since the global effect of the biomass composition on the outcome of the simulations is negligible (as shown above), we considered the use of the original biomass equation to be justified.

**Table 4:** Comparison of the *in silico* predicted growth yields (in g_DW_⋅g_Element_^−1^) with experimental continuous culture data.

Limiting Element	Yield – Experimental	Yield – Model
C	0.47	0.61
N	5.74	6.67
P	84.95	34.92
S	268.75	130.18

### Analysis of Blocked Reactions: The Quest for Completeness

As described above, iJP815 contains 289 unconditionally (i.e., not dependent on external sources) blocked reactions (that is, reactions unable to function because not all connections are made), corresponding to 33% of the metabolic network. In previously published genome-scale metabolic reconstructions, the fraction of blocked reactions varies between 10 and 70 percent [48]. Blocked reactions occur in reconstructions mostly due to knowledge gaps in the metabolic pathways. Accordingly, the blocked-reactions set can be divided into two major groups; (1) reactions with no connection to the set of non-blocked reactions, and (2) reactions that are either directly or indirectly connected to the operating core of the *P. putida* model. The first group of reactions includes members of incomplete pathways that, with increasing knowledge and further model refinement, will gradually become connected to the core. This subset comprises 108 reactions (35% of blocked reaction set). The second group of reactions comprises also members of incomplete pathways, but many of them belong to pathways that are complete but that lack a transport reaction for the initial or final compound. Examples of pathways lacking a transporter are the degradation of fatty acids and of propanoate.

In addition, there could exist compounds whose production is required only in certain environmental conditions, e.g., under solvent stress, and as such are not included in generic biomass equation. Pathways synthesizing compounds that are not included in the biomass equation but that likely are conditionally required include the synthesis of thiamine, various porphyrins and terpenoids. In this case, reactions involved exclusively in the production of such compounds would be blocked if no alternative outlets exist for those pathways. Allowing a non-zero flux through these reactions would require inclusion into biomass of the conditional biomass constituents, which in turn would require having various biomass equations for various conditions. This level of detail, however, is beyond the scope of our initial metabolic reconstruction and investigation.

The high number of blocked reactions in iJP815 clearly indicates that there are still vast knowledge deficits in the model and, thus, in the underlying biochemical and genomic information. Since a genome-scale metabolic model seeks to incorporate all current knowledge of an organism's metabolism, these reactions are integral elements of the metabolic reconstruction and of the modeling scaffold, even if they are not able to directly participate in steady state flux studies. Therefore, the inclusion of these reactions in the model provides a framework to pin-point knowledge gaps, to include novel information as it becomes available and to subsequently study their embedding and function in the metabolic wiring of the cell.

### How *P. putida* Allocates Its Resources: Evaluating the Prediction of Internal Flux Distributions

The assessment performed as described above by means of high-throughput phenotyping assays, growth experiments and continuous cultivations, has shown that the model is coherent and that it captures the major metabolic features of *P. putida*. We subsequently used the model to probe the network and to ascertain the distribution of internal fluxes and properties such as network flexibility and redundancy of particular reactions. To this end, we predicted the distribution of reaction fluxes throughout the central pathways of carbon metabolism by flux variability analysis (FVA), and compared the simulations to internal fluxes computed from experimentally obtained ^13^C data in *P. putida* [49],[50].

### Optimal FVA

**Figure 4:**
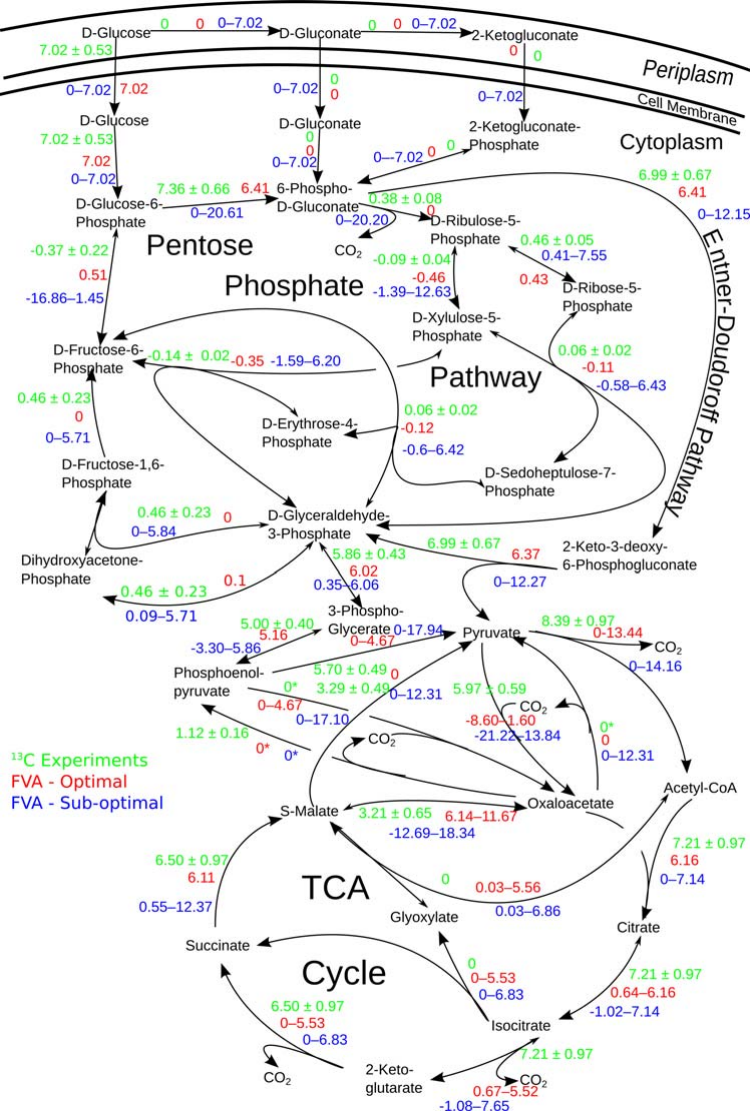
Comparison of FVA calculations with ^13^C experimental flux data. The explanation of color codes is given in the figure. “0*” means that the reaction is not included in the particular metabolic network; double-headed arrows depict reversible reactions, the bigger head shows direction of the positive flux.

Genome-scale metabolic networks are, in general, algebraically underdetermined [41]. As a consequence, the optimal growth rate can often be attained through flux distributions different than the single optimal solution predicted by FBA simulations. Therefore we used flux variability analysis (FVA) to explore the network, as this method provides the intervals inside which the flux can vary without influencing the value of the growth yield (if the flux of the reaction cannot vary then the range is limited to a single value) [41]. The results of the simulations are given in Figure 4. As isotopic (^13^C) measurements are not able to distinguish which glucose uptake route is being used by *P. putida*, all the fluxes in the ^13^C experiment and in the FVA simulations were computed assuming that glucose is taken up directly into the cell. For the precise description of the network models used in this comparison (i.e., FBA/FVA vs. ^13^C-Flux analysis) see Text S1 and Text S2 (sections “Comparison of FVA analyses with ^13^C flux measurement data”).

Figure 4 shows that the predictions (in red) generally agree well with the measurements (in green) throughout the network, as most of the ^13^C values fall within the FVA intervals, where intervals were predicted, or both values are close to each other (in absolute values), when a single value was predicted. As *P. putida* lacks phosphofructokinase, glucose can be converted to pyruvate (the entry metabolite of TCA cycle) via the pentose phosphate (PP) or the Entner-Doudoroff (ED) pathways. The ED pathway is energetically more efficient and the ^13^C measurements indicate that KT2440 uses it preferentially over the PP pathway. Therefore, the FVA yields locally single flux values rather than intervals, which reflects the relative rigidity of this part of the network. In contrast, the energy generating part of the central metabolic network (the TCA cycle and its vicinity) exhibits greater flexibility, as illustrated by the broad flux intervals. Firstly, the conversion of phosphoenylpyruvate into pyruvate can proceed either directly or via oxaloacetate, although the bacterium appears to use the direct route (the ^13^C-model assumes, in fact, only the direct route; see Text S1, section “Comparison of FVA analyses with ^13^C flux measurement data”). Secondly, the conversion of malate to oxaloacetate may also occur directly or via pyruvate. The ^13^C flux measurements indicate that the bacterium uses the indirect route in addition to the direct one although, according to the FVA, the indirect route is energetically less efficient. Interestingly, our model suggests also that the glyoxylate shunt could be used interchangeably with full TCA-cycle without any penalty on growth yield. However, as the glyoxylate shunt is inactivated in many bacterial species via catabolite repression upon glucose growth [51], it is possible that this alternative is not used in *P. putida.*

### Discrepancies between Model Predictions and Measurements

Despite the general agreement between *in silico* predictions and ^13^C measurements, there still exist a number of discrepancies. For instance, the ^13^C-experiments suggest that the bacterium utilizes the portion of glycolysis between triose-3-phosphate and d-fructose-6-phosphate in the gluconeogenic direction, which is not energetically optimal and as such is not captured in standard FBA (or FVA) simulations. This illustrates one of the possible pitfalls of FBA, which per definition assumes perfect optimality despite the fact that microorganisms might not necessarily allocate their resources towards the optimization function assumed in analysis, and in some cases may not operate optimally at all [52],[53]. Another group of differences concentrates around the pentose phosphate pathway (PPP), although these are relatively minor and are likely due to differences in the quantities of sugar diverted toward biomass in the ^13^C model vs. iJP815. A third group of differences revolves around pyruvate and oxaloacetate, whereby the *in vivo* conversion of malate to oxaloacetate shuttles through a pyruvate intermediate rather than directly converting between the two. The last area where discrepancies exist between *in silico* and ^13^C data is in the TCA cycle, around which the flux is lower in FVA simulations than in the experiment. This suggests that the *in silico* energetic requirements for growth (maintenance values) are still too low when compared to *in vivo* ones, as the main purpose of the TCA cycle is energy production.

### Suboptimal FVA

To investigate further these differences, we carried out a suboptimal FVA (Figure 4, blue values), allowing the production of biomass to range between 90 and 100% of its maximum value. In this suboptimal FVA experiment, the ^13^C-derived fluxes fall between FVA intervals for every flux value in the ^13^C network. To filter out artifacts, we re-did all FVA computations using the structure of the network used in the ^13^C-experiment and found no major differences (see Figure S3). We also assessed the influence of the biomass composition on the distribution of internal fluxes and network structure and found that this was negligible on both accounts (see Text S2, section “Evaluation of biomass equation composition on the outcome of FBA/FVA simulations” and Figure S4). The results show that, in principle, the bacterium can use all the alternatives described above and that the penalty on the growth yield is minimal. While this analysis validates the FVA simulation results, the wide breadth of the intervals (i.e., the mean ratio of interval width to mean interval value exceeds three), suggests that the (mathematical) under-determination of central metabolism can be quite high, and indicates that there exist multiple sub-optimal solutions across the network and that is thus difficult to predict exact internal flux and to “pin-point” a particular solution. These results reflect the essence of constraint-based modeling and FBA, which provide only a space of possible flux distributions and not exact values. Therefore, deductions from results of FBA simulations have to be made with great care. This underscores the notion that constraint based modeling should be seen more as navigation framework to probe and explore networks rather than as an exact predictive tool of cellular metabolism.

### Gauging the Robustness of the Network

#### Essentiality of genes and reactions

**Figure 5:**
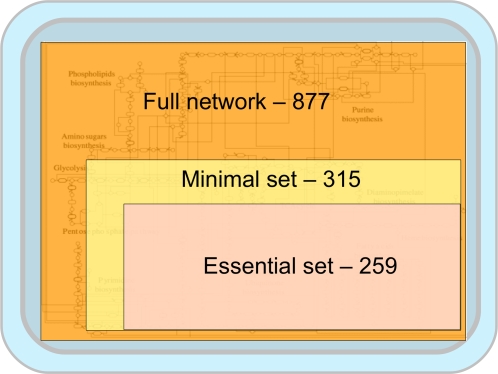
Interdependency between the metabolic network, the minimal set and the set of essential reactions. The set sizes are given for glucose growth conditions.

To assess the robustness of the metabolic network to genetic perturbations (e.g., knock-out mutations), we carried out an *in-silico* analysis of the essentiality of single genes and reactions, which enabled us to identify the most fragile nodes of the iJP815 network. Reaction essentiality simulations were performed by systematically removing each reaction from the network and by assessing the ability of the model to produce biomass *in silico* via FBA in minimal medium with a sole carbon source (glucose and acetate). Gene essentiality was assessed by: (i) identifying for each gene the operability of the reaction(s) dependent on this gene, (ii) removing from the network the reactions rendered inoperative by the deletion of that particular gene, and (iii) determining the ability of the model to produce biomass in the same manner as for the reaction essentiality tests. Additionally, we estimated for both carbon sources the smallest possible set of reactions able to sustain *in silico* growth, in order to estimate the number of reactions necessary for biomass synthesis in minimal medium (minimal set). This set encompasses both all reactions that are essential (including those essential regardless of the medium and those ‘conditionally essential’) and the minimal number of non-essential reactions that, together, are able to provide *in silico* growth (see Figure 5). These conditionally essential reactions can be used as a reference for identifying sections of metabolism for which alternative pathways exist. For both glucose and acetate, the minimal sets encompassed approximately 315 reactions (Table S5). This estimate is consistent with values obtained for other bacteria [54].

The sets of essential reactions consist of 259 and 274 reactions for glucose and acetate conditions respectively, constituting 82 and 86 percent of the minimal set. These numbers indicate that most of the crucial metabolic routes are not duplicated at the level of metabolic network structure. The set of essential reactions under glucose growth is a subset of that under acetate, suggesting that the growth on glucose is more resistant to perturbations (as the smaller number of reactions mean less fragility points in the network). The reactions belonging exclusively to the acetate minimal set are mostly members of glyconeogenic pathway, with ATP synthase, the reactions constituting the glyoxylate shunt, and acetate transport reactions being the exceptions. The inessentiality of ATP synthase under glucose and essentiality of the glyoxylate shunt under acetate conditions are not surprising and similar effects have been reported in *E. coli* [55]–[57].

The reactions belonging to the non-essential part of the minimal set are mostly members of central metabolic pathways (PPP, TCA cycle, and Pyruvate metabolism), which emphasizes the importance of these pathways for the operation of the metabolism and is in agreement with observations made in other bacteria [58].

#### Isoenzymes

The metabolic robustness of an organism may also be provided at the genetic level through genes coding for isozymes. Data on gene and reaction essentiality provide insights into this phenomenon. We utilized FBA to generate a list of *in silico* essential gene predictions, including 153 and 159 genes under minimal glucose and acetate growth respectively, in order to determine how gene/pathway redundancy affects network robustness. These values may seem low when compared to the size of the predicted essential reactions sets (259 and 274 reactions for glucose and acetate growth, respectively). However, it must first be noted that each essential reaction set contains about 25 (26 and 27 for the glucose and acetate essential set, respectively) non gene-associated reactions, and that elucidating the genes catalyzing these reactions would increase substantially the number of *in silico* essential genes. Further, approximately 20% of each minimal set (78 and 84 genes under glucose and acetate conditions, respectively) consists of essential reactions that can be catalyzed by two or more isozymes and thus are essential at the metabolic network level but not at the genetic level. In contrast to non-essential reactions in the minimal set, these reactions essential at the metabolic network level but not at the genetic level are not clustered in particular metabolic pathways but are rather spread throughout the entire metabolic network. Altogether, these results indicate that for about 40% of the reactions required to produce biomass, there are alternative at either the genetic or the metabolic network level.

This analysis highlights the limitations of possible interventions aimed at reshaping the flux distributions, because these can be applied only to reactions that are not essential (since the inactivation of an essential reaction yields a lethal phenotype). Identification of reactions catalyzed by multiple enzymes shows which reactions may be best avoided when planning mutational strategies as their inactivation may pose additional technical problems, by requiring production of multiple knock-outs.

#### Flexibility of flux distributions

To further investigate these conclusions, we determined the flexibility of fluxes over particular reactions as a measure of metabolic network flexibility during biomass production. We found that the variability of fluxes is similar under either glucose or acetate growth, but that acetate growth instills a slightly higher rigidity to the metabolic network (as observed above). We observed also that the flux of more than a half of the reactions can vary to some degree without influencing biomass output. We next analyzed the pathway-distribution of reactions exhibiting variable flux, and found that biosynthetic pathways are in general more rigid (i.e., the fraction of reactions with flexible flux is relatively lower) than other pathways. This rigidity might reflect the essentiality of these pathways modules for the survival of the cell (See sections “Analysis of flexibility of the flux over particular reactions” in the Text S1 and Text S2, Figure S5, Table S3 and Table S5). A further measure to ascertain network flexibility was the assessment of pairwise couplings between the reactions via Flux Coupling Finder (see Text S1 and Text S2, sections “Flux Coupling Finder” and Figure S6). This analysis indicated that for 90% of the reactions that are unblocked in a given condition, at least one other reaction exists whose flux is proportionally coupled to the flux of the first reaction, and therefore that the great majority of reactions can be inactivated through inactivation of some other reaction. This analysis is helpful in optimizing mutational strategies as it pin-points alternative mutations that exhibit equivalent outcomes.

### Prediction of Auxotrophic Mutations and Model Refinement

Assessment of network models through comparison of *in silico* growth-phenotypes with the growth of knock-out strains is a powerful way to validate predictions. This has been done in a number of studies for which knock-out mutant libraries were available [59],[60]. As there is currently no mutant library for *P. putida*, we tested gene knock-out predictions with a set of *P. putida* auxotrophic mutant strains created in our laboratory that are incapable of growth on minimal medium with acetate as the sole carbon source. First we compared whether the corresponding *in silico* mutants followed the same behavior (lack of growth on minimal medium with acetate, where zero biomass flux during FBA corresponded to a no-growth phenotype). This comparison was performed only for strains whose knocked-out gene is included in iJP815. Thirty-eight out of the 51 strains tested did not grow *in silico* (Table S4). Of the remaining 13 false positives (i.e., those growing *in silico* but not *in vivo*), four (PP1470, PP1471, PP4679, and PP4680) are mutated in genes considered non-essential *in silico* due to “weakly annotated” gene putatively encoding redundant isozymes. In the case of PP5185 (coding for *N*-acetylglutamate synthase), its essentiality is removed by PP1346 (coding for bifunctional ornithine acetyltransferase/*N*-acetylglutamate synthase protein), which is not only an isozyme of PP5185 (the *N*-acetylglutamate synthase function) but which also catalyses a reaction (ornithine acetyltransferase) that produces *N*-acetyl-l-glutamate (the product of *N*-acetylglutamate synthase) and thus renders the activity of PP5185 redundant. It appears either that this is a mis-annotation or that the enzyme is utilized only under different conditions.

In addition, PP0897 (*fum*C) seems to have two paralogues (PP0944, PP1755) coding for isoenzymes of fumarate hydratase, but since the mutant in PP0897 does not grow auxotrophically, they are either non functional or mis-annotated. The enzyme complex that is composed of proteins expressed from the genes knocked-out in the two false positives PP4188 and PP 4189 catalyzes the decarboxylation of α-ketoglutarate to succinyl-CoA in the TCA cycle, concurrently producing succinyl-CoA for anabolic purposes. In the model, this functionality is not needed as this part of the TCA cycle can be circumvented by the glyoxylate shunt, whereas succinyl-CoA can be produced by reverse operation of succinate-CoA ligase. Restricting this reaction to be irreversible renders both genes essential. This altogether suggests that either the succinate-CoA ligase is irreversible or the glyoxylate shunt is inactive. The latter solution is, however, impossible, due to the essentiality of the glyoxylate shunt upon growth on acetate.

The false positive PP4782 is involved in thiamine biosynthesis. This cofactor is not included in the biomass, which is why the gene is not *in silico* essential. This suggests thus that the *in-silico P. putida* biomass reaction should be enriched with this cofactor. The remaining false positives (PP1768, PP4909, PP5155) are involved in the serine biosynthesis pathway. We found experimentally that mutants in these genes can grow on acetate if the medium also contains l-serine. These genes can be rendered *in silico* essential by setting glycine hydroxymethyltransferase to operate only unidirectionally from l-serine to glycine. The operation of this enzyme, however, is required for growth of the bacterium on glycine, which is possible; though very slow (results not shown). One of these genes (PP5155) has also a weakly annotated isozyme (PP2335). We found out as well that several of the mutants (PP1612, PP4188-9, PP4191-4) grow *in silico* on glucose, which we confirmed experimentally (results not shown). Altogether, these experimental results assisted us in improving the accuracy of the model.

Albeit limited to a relatively small mutant set, this analysis shows that while constraint-based models are not always able to predict exact flux values, they are very useful in the identification of essential reactions and, through the GPRs, the genes responsible for their catalysis. This enables identification of vulnerable points in the metabolic network.

### Model Application—Production of Polyhydroxyalkanoates from Nonalkanoates

To illustrate the utility of a genome-scale model for metabolic engineering, we used iJP815 to predict possible improvements to an industrially relevant process; namely, the production of polyhydroxyalkanoates (PHAs) from non-alkanoic substrates for biomedical purposes [61]–[63]. As the production of PHAs uses resources that would be otherwise funneled towards growth, increasing *in silico* PHA production would decrease the growth. Consequently, in classic optimization-based approaches (e.g., FBA), no PHA production would be predicted while optimizing for growth yield. The aim was thus to increase the available pool of the main precursor of PHAs—Acetyl Coenzyme A (AcCoA). This approach was based on the observation that inactivation of isocitrate lyase (ICL) enhances the production of PHAs in *P. putida* due to increased availability of AcCoA that is not consumed by ICL [64]. We therefore searched for other possible intervention points (mutations) in the metabolic network that could lead to the accumulation of AcCoA. This analysis was performed through application of a modified OptKnock approach [28], which allowed for parallel prediction of mutations and carbon source(s) that together provide the highest production of the compound of interest.

**Figure 6:**
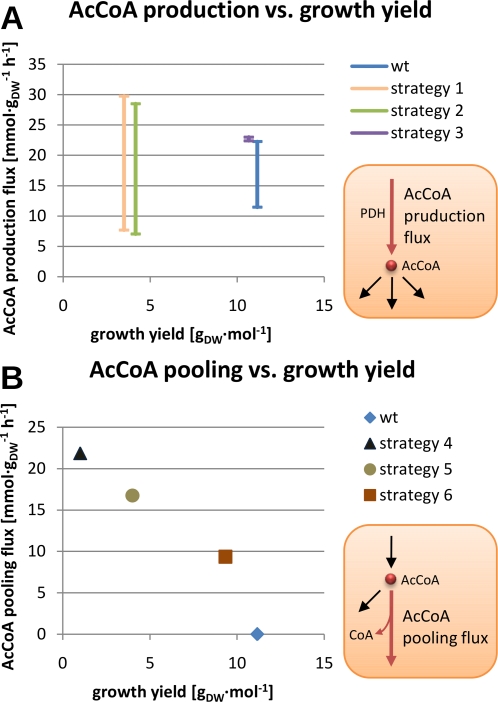
Mutational strategies for increased PHA production. This figure highlights 6 strategies suggested by the modified optknock approach for increased production of AcCoA, a precursor for polyhydroxyalkanoates. (A) AcCoA production ranges vs. growth yield of *in silico* strains developed using the ‘AcCoA production’ strategy. (B) AcCoA pooling versus growth yield of *in silico* strains developed using the ‘AcCoA pooling’ strategy.

Two main methods were employed to model a cellular pooling of AcCoA. The first was the maximization of AcCoA production by pyruvate dehydrogenase (PDH). In the second, an auxiliary reaction was introduced that consumed AcCoA (concurrently producing CoA, to avoid cofactor cycling artifacts) and that would represent the pooling of AcCoA (Figure 6A and 6B, insets). It is noteworthy that the value of ‘AcCoA production’ predicted by the first method includes AcCoA that is then consumed in other reactions (some of which will lead towards biomass production for instance), whereas the value of ‘AcCoA pooling’ predicted by the second method includes only AcCoA that is taken completely out of the system, and therefore made available for PHA production but unusable for growth or other purposes. Therefore, only with the first method (AcCoA production) can AcCoA fluxes and growth rates be compared directly with the wild-type AcCoA flux and growth rate, as the second method (AcCoA pooling) will display lower values for AcCoA fluxes and growth rates but will avoid ‘double counting’ AcCoA flux that is shuttled towards growth, and therefore is not available for PHA production (see plots in Figure 6A and 6B).

To create the *in silico* mutants, we allowed the OptKnock procedure to block a maximum of two reactions, which corresponds, experimentally, to the creation of a double mutant. To avoid lethal *in silico* strains, the minimal growth yield was limited to a value ranging between 0.83 and 6.67 g_DW_⋅mol_C_^−1^, corresponding to about 5 and 40 percent of maximum growth yield, respectively.

Six mutational strategies suggested by this approach are presented in Table 5. The first three were generated by the AcCoA production method, and the last three were generated by the AcCoA pooling method. The results provide a range of options for possibly increasing AcCoA production, some of which constrain growth more than others (see Figure 6A and 6B).

**Table 5:** Summary of the characteristics of the *in silico* strains generated in the procedure of optimization of the PHA production.

Strain	Blocked Enzymatic Activity	Loci To Be Blocked	Carbon Source(s)	AcCoA Production [mmol g_DW_^−1^·h^−1^]		Growth Yield [g_DW_·mol_C_^−1^]	
				Min	Max	Limit	Sim
WT	WT	WT	l-Serine	11.47	22.26	0.83	11.16
1	Triose-phosphate isomerase	PP4715	d-Fructose	7.7	29.74	0.83	3.5
	6-Phosphoglucono lactonase	PP1023					
2	Glucose dehydrogenase (membrane)	PP1444	d-Glucose	7.05	28.51	0.83	4.17
	6-Phosphoglucono lactonase	PP1023					
3	Isocitrate dehydrogenase	PP4011 or PP4012	l-Serine	22.41	23.01	6.66	10.67
	Formate dehydrogenase	PP0490 or PP0491					
		PP2183 or PP2184 or PP2185 or PP2186					
4	Citrate synthase	PP4194	l-Valine	21.85		0.83	1.00
	2-Methylcitrate dehydratase	PP2338					
5	Glycine hydroxymethyl transferase	PP0322	l-Leucine, l-lysine, l-phenyl­alanine	16.75		3.33	4.00
		PP0671					
	Citrate synthase	PP4194					
6	Glycine hydroxymethyl transferase	PP0322	l-Leucine, l-isoleucine	9.35		6.66	9.33
		PP0671					
	Citrate synthase	PP4194					

One promising hypothesis (strategy 2) generated by the AcCoA production method predicted that a double-mutant devoid of 6-phosphogluconolactonase (*pgl*/PP1023) and periplasmatic glucose dehydrogenase (*gcd*/PP1444), would produce 29% more AcCoA than the wild type growing on glucose as a carbon source (Figure 6A). As we are currently still in the process of generating this mutant, we were not yet able to test the prediction. Another promising hypothesis (strategy 1) included knocking-out triose phosphate isomerase (*tpiA*/PP4715). As the mutant for *tpiA* was generated in this work, we tested whether it is able to grow on the predicted carbon source (d-fructose), but the observed growth was very weak (only very small colonies grew on agar plates after three days). This suggests that growth might be too inhibited by this strategy for it to be of great use.

One strategy suggested by the AcCoA pooling method (strategy 4) called for knocking out 2-methylcitrate dehydratase (*prpD*/PP2338) and citrate synthase (*gltA*/PP4194), and supplying *P. putida* with valine. Using this strategy, AcCoA pooling could theoretically reach 21.9 mmol⋅g_DW_^−1^⋅h^−1^, but at a severe expense in bacterial growth (Figure 6B). The other strategies suggested by the AcCoA pooling method highlight a somewhat linear tradeoff between growth and AcCoA pooling, which could be investigated experimentally to determine how much growth disruption is acceptable in a bioengineered production strain of *P. putida* (Figure 6B).

These strategies illustrate the possible approaches to optimizing production of a non-growth associated compound, and highlight the need for further experimental work to assess the performance of this approach.

## Discussion

A primary value of genome-scale metabolic models is their ability to provide a holistic view of metabolism allowing, for instance, for quantitative investigation of dependencies between species existing far apart in the metabolic network [20]. Once experimentally validated, these models can be used to characterize metabolic resource allocation, to generate experimentally testable predictions of cell phenotype, to elucidate metabolic network evolution scenarios, and to design experiments that most effectively reveal genotype-phenotype relationships. Furthermore, owing to their genome-wide scale, these models enable systematic assessment of how perturbations in the metabolic network affect the organism as a whole, such as in determining lethality of mutations or predicting the effects of nutrient limitations. Since these multiple and intertwined relationships are not immediately obvious without genome-scale analysis, they would not be found during investigation of small, isolated circuits or genes as is typical in a traditional reductionist approach [65],[66].

We present here a genome-scale reconstruction and constraint-based model of the *P. putida* strain KT2440, accounting for 815 genes whose products correspond to 877 reactions and connect 886 metabolites. The manually curated reconstruction was based on the most up-to-date annotation of the bacterium, the content of various biological databases, primary research publications and specifically designed functional genomics experiments. New or refined annotations for many genes were suggested during the reconstruction process. The model was validated with a series of experimental sets, including continuous culture data, BIOLOG substrate utilization assays, ^13^C flux measurements and a set of specifically-generated mutant strains. FBA and FVA were used to ascertain the distribution of resources in KT2440, to systematically assess gene and reaction essentiality and to gauge the robustness of the metabolic network. Hence, this work represents one of the most thorough sets of analyses thus far performed for an organism by means of constraint-based modeling, providing thereby a solid genome-scale framework for the exploration of the metabolism of this fascinating and versatile bacterium. However, since this modeling endeavor relies upon a number of approximations, the limits, potential and applicability of the analysis must be clearly identified and defined. We address these points below.

Altogether, our results and analyses show that the model accurately captures a substantial fraction of the metabolic functions of *P. putida* KT2440. Therefore, the model was used to generate hypotheses on constraining and redirecting fluxes towards the improvement of production of polyhydroxyalkanoates, which are precursors for industrially and medically important bioplastics. This is, to our knowledge, the first reported application of constraint-based modeling to direct and improve the yield of a compound of which the production is not directly coupled to the growth of the organism. This opens up novel areas of application for the constraint-based approach. Our approach, based on the OptKnock algorithm, allows for both prediction of mutants with desirable properties and identification of conditions that support the expression of these properties.

Notwithstanding the generally good agreement between experimental results and simulations of our model, several of the discrepancies encountered reflect pitfalls inherent to constraint-based modeling that go beyond the scope of our study:

Firstly, the high number of blocked reactions and the mismatches with the BIOLOG data show that there are still many areas of the metabolism that require thorough exploration. The genes encoding transport-related are particularly relevant, as for most of them, neither the translocated compound nor the mechanism of translocation is known. Furthermore, it should be highlighted that the genome still has 1635 genes annotated as “hypothetical” or “conserved hypothetical”, more than 800 genes annotated as putative, and over 800 for which the functional annotation gives no information beyond the protein family name. It is thus likely that a fraction of the hypothetical and non-specifically annotated genes in the current *P. putida* annotation are responsible for unknown metabolic or transport processes, or that some might code for proteins that add redundancy to known pathways. This observation is common to all genomes sequenced so far and illustrates a major hurdle in the model building process (and hence, its usefulness) that can be overcome only through extensive studies in functional genomics.

Secondly, although we carefully constrained the *in silico* flux space through FBA and FVA and obtained distribution spaces roughly consistent with those experimentally determined via ^13^C- flux analysis, these approaches are inherently limited as they assume growth as a sole metabolic objective and ignore any effects not explicitly represented in a constraint-based metabolic model. It has been shown that FBA using objective functions other than growth can improve predictive accuracy under certain conditions [53]. Kinetic limitations also may play a very important role in determining the extent to which a particular reaction or pathway is used. Teusink et al. [52] showed that in the case of *L. plantarum* these factors may lead to false predictions.

Thirdly, the reconstruction includes causal relationships between genes and reactions via gene-protein-relationships (GPRs) but it lacks explicit information regarding gene regulation. The regulation of gene expression causes that there are many genes in the cell that are expressed only under certain growth conditions. Therefore, the *in silico* flux space is generally larger than the true *in vivo* flux space of the metabolic network. This, in turn, may influence the robustness of the metabolic network and the essentiality of some reactions and genes. The lack of regulatory information and of the genetic interactions involved is likely to be one of the causes for faulty predictions of the viability of mutant strains. Adding this information will be an important step in the further development and improvement of the accuracy of the reconstruction.

Fourthly, although our analyses indicated that growth yield is relatively insensitive to changes in biomass composition, these analyses also suggest that factors other than the structure of the metabolic network play an important role in defining the relationship between the growth yield and environmental conditions. The prediction of the exact growth yield requires the precise measurement of maintenance values, which may vary substantially from one condition to the other [44]–[46]. As the maintenance accounts for 10–30% of the total carbon source provided in unstressed conditions, this may set a limit to the accuracy of the growth yield predictions.

To enhance the usefulness and predictiveness of the model, several avenues could be followed in the future. Firstly, additional constraints can be overlaid on the network to reduce the space of possibilities and increase the accuracy of predictions. In addition to specific knowledge of particular enzymatic or transport processes, such constraints are best based on high-throughput experimental evidence such as transcriptomic and proteomic data, which are instrumental in expanding genotype-phenotype relationships in the context of genome-scale metabolic models [67]. Microarray experiments have guided the discovery of metabolic regulons, and usage of microarray and proteomic data to constrain metabolic models has improved model accuracy for other systems [23]. Secondly, *P. putida* provides a good opportunity for incorporating kinetic information into a genome-scale model as there are various kinetic models available and under development for small circuits in *P. putida* [68]–[71]. Incorporating data from these models into the genome-scale reconstruction would provide insights into the relationships of isolated metabolic subsystems within the global metabolism. This synthesis would also improve the flux predictions of the global model, particularly in areas where current FBA-based predictions methods fail due to their inherent limitations.

Experimental validation of a genome-scale model is an iterative process that is performed continuously as a model is refined and improved through novel information and validation rounds. In this work, we have globally validated iJP815 as well as specific parts thereof by using both up-to-date publicly available data and data generated in our lab, but there will be always parts of the model that include blocked reactions and pathways that will require further, specific validation. As more knowledge becomes available from the joint efforts of the large *P. putida* community (e.g., http://www.psysmo.org), focus will be put on these low-knowledge areas for future experimental endeavors. We anticipate that this model will be of valuable assistance to those efforts.

The metabolic reconstruction, the subsequent mathematical computation and the experimental validation reported here provide a sound framework to explore the metabolic capabilities of this versatile bacterium, thereby yielding valuable insights into the genotype-phenotype relationships governing its metabolism and contributing to our ability to exploit the biotechnological potential of pseudomonads. By providing the means to examine all aspects of metabolism, an iterative modeling process can generate logical hypotheses and identify conditions (such as regulatory events or conditional expression of cellular functions) that would reconcile disagreements between experimental observations and simulation results. Through a detailed *in silico* analysis of polyhydroxyalkanoate production, we show how central metabolic precursors of a compound of interest not directly coupled to the organism's growth function might be increased via modification of global flux patterns. Furthermore, as the species *Pseudomonas putida* encompasses strains with a wide range of metabolic features and numerous isolates with unique phenotypes, the reconstruction presented provides a basic scaffold upon which future models of other *P. putida* strains can be built with the addition or subtraction of strain-specific metabolic pathways. Due to its applicability across the numerous *P. putida* strains iJP815 provides a sound basis for many future studies towards the elucidation of habitat-specific features, bioremediation applications and metabolic engineering strategies with members of this ubiquitous, metabolically versatile and fascinating genus.

## Materials and Methods

### Constraint-Based Models

The *P. putida* model we present was built using a constraint-based (CB) approach. A constraint-based model consists of a genome wide stoichiometric reconstruction of metabolism and a set of constraints on the fluxes of reactions in the system [19],[20],[24]. The reconstruction represents stoichiometry of the set of all reactions known to act in metabolism of the organism, which can be determined in large part from genomic data since most cellular reactions are catalyzed by enzymes. Thus the model does not require any knowledge regarding the kinetics of the reactions, and the requisite thermodynamic knowledge is limited to the directionality of reactions.

In addition to the reactions, the model includes a set of genes tied via Boolean logic to reactions that their protein products catalyze, which allows for accurate discrimination of the effects of genetic perturbations such as knockouts [33],[72]. These Boolean rules together form the gene-protein-reaction relationships (GPRs) of the metabolic reconstruction [33].

The second part of the CB-model, namely the constraints, constitutes a set of rules that narrow down the interval within which the flux of particular reaction must lie. These constraints rest upon physico-biological knowledge. One of them, the information regarding reaction directionality, has already been mentioned above. Another constraint that is widely applied in biological systems is the Pseudo-Steady-State Assumption (PSSA) [73], which states that a concentration of a chemical compound stays constant over the simulated time frame. The reactants to which this constraint is applied are usually called internal compounds, and in biological models correspond to the chemical substances located inside the cell or its compartments. Remaining substances, external compounds, correspond to species that can be taken up or secreted and thus exchanged with the environment. Other types of constraints are top and bottom limits that correspond to catalytic capabilities of the enzymes. More detailed description of constraint based modeling approach can be found in [74] and the Text S1, section “Constraint based models—mathematical explanation”.

### Analysis Methods

#### Flux balance analysis

Flux balance analysis (FBA) is a primary method for analysis of constraint-based models. Generally, a constraint based model of metabolism represents an underdetermined system, i.e., one in which a range of flux distributions are mathematically possible. FBA narrows the flux possibilities by determining a point in closed flux space that maximizes a certain linear combination of fluxes. [75]. FBA poses a linear programming (LP) problem and thus a global maximum always exists, provided that the problem is feasible (i.e., there exists at least one combination of fluxes which fulfills all the constraints). Using the matrix notation the FBA problem can be stated as following:
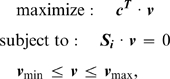
where ***S*** is the stoichiometric matrix containing reaction stoichiometry information, ***v*** is a vector of all reaction fluxes in the system, ***v***_min_ and ***v***_max_ represent minimum and maximum constraints on reaction fluxes, respectively, and ***c^T^*** is a vector containing coefficients for each flux that is to be maximized (for more detail on FBA, refer to [76]).

FBA optimization yields an optimal value for the objective along with a flux value for every reaction belonging to the metabolic network. Commonly, FBA is used to predict maximal growth or metabolite production yields. Cell growth is simulated by the flux over a special ‘Biomass’ reaction that consumes precursors of cellular components (amino acids, lipids, dNTPs, NTPs, cofactors) and produces a virtual unit of cell biomass. Maximization of this flux is usually set as the FBA objective. This procedure assumes that organisms have been shaped by the evolution towards growth maximization, an assumption that has been validated under a variety of conditions [77].

#### Flux variability analysis

Metabolic networks of living organisms are usually considerably underdetermined [78]–[80]. The size of the mathematically allowed flux space can vary depending upon the network structure and the constraints. Flux variability analysis (FVA) is a method that allows for rough top estimation of the flux space for a given FBA optimization [41]. FVA computes for each reaction an interval of values inside of which the flux of the reaction can change without influencing value of the objective function, provided that other fluxes are allowed to vary freely within their constraints.

It is often the case that cells do not operate perfectly optimally when FBA simulations are compared to real data. Therefore, a variant of the FVA approach called suboptimal FVA [41] is sometimes informative, wherein instead of fixing the objective to its optimal value from the initial FBA run (as in standard FVA), the objective value is allowed to vary within a predetermined limit. For every suboptimal FVA presented in this paper the objective lower limit was chosen at 90% of the initial objective value (assuming that FBA maximized the objective).

### OptKnock

OptKnock is an approach for identification of mutations that selectively increase production of a certain compound of interest, assuming that the mutant would optimize for the same quantity as the wild type (e.g., growth yield) [28]. OptKnock points out reactions (and genes, through GPR logic) that must be blocked in order to maximize a linear combination of target fluxes (outer objective) while simultaneously maximizing for the cell's assumed objective (growth yield; inner objective). OptKnock poses a bi-level optimization approach that is solved via Mixed-Integer Linear Programming (MILP). Further details can be found in Text S1, section “OptKnock – mathematical formulation” and [28].

#### OptKnock—modification

In order to enable the choice of the carbon source(s) the original OptKnock procedure was modified as follows:

1. A virtual reaction, with limited flux, was created that sourced the virtual compound “vcarbon”
2. For each carbon source a virtual irreversible reaction that converted the compound *“vcarbon”* into the respective carbon source was added to the model. The stoichiometry of this virtual reaction corresponded to the number of carbon atoms in the carbon source, e.g.:
  6 *vcarbon* → *d-glucose*.
3. For each of those reactions (*v_j_*) a binary variable (*z_j_*) defining its activity was created and following constraint was added to the model: *v_j_* ≤ *v_j_* ^max^·*z_j_*, where the *v_j_* ^max^ was set to value high enough, so that the whole “vcarbon” could be consumed by each reaction.

This modification allows for the choice of one or more carbon sources that, together with the mutation set identified by OptKnock, provide the highest objective.

### Identification of Minimal Growing Reaction Set

The minimal growing set was identified using a Mixed Integer Linear Programming (MILP) approach, by modifying original FBA LP problem. For every non-blocked and non-essential reaction a binary variable was added that reflects the activity of the reaction. When the binary variable takes value of 1 the corresponding reaction is virtually unlimited (or limited by rules of original LP problem). When the variable is set to 0 the corresponding reaction is blocked (non-zero flux is impossible). This was achieved by adding a following set of equations to the original LP problem:
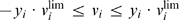
for reversible reactions, and
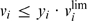
for irreversible reactions. In order to assure that growth was not overly restricted, a minimal flux value was established for the biomass reaction. We set the lower limit on biomass flux to 0.05 when the supply of carbon source was 60 mmol_C_·g_DW_^−1^h^−1^, which corresponds to growth yield of 0.07 g_DW_·g_C_^−1^, 16 times lower than the wild type. The objective of the problem was set to minimize the sum of all binary variables *y_i_*: 
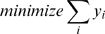
This method searches for a minimal set that is able to sustain growth greater than or equal to to the minimal growth requirement.

### Metabolic Network Reconstruction

The main sources of information regarding the composition of the metabolic network of *Pseudomonas putida* KT2440 were various biological databases. Most of the information came from the Kyoto Encyclopedia of Genes and Genomes (KEGG) [35],[81] and Pseudomonas Genome Database (PGD) [82]. Information regarding *P. putida* contained in these two databases is mainly based on the published genome annotation of the bacterium [14], so there is a large overlap between them. Additionally, substantial information was taken from the BRENDA database, which catalogs reaction and enzyme information [83]. This all was augmented with knowledge coming directly from primary research publications (see Text S3). The reconstruction process was performed in an iterative manner, i.e., by adding or removing reactions from the model in between rounds of model testing. First, reaction information for *P. putida* was collected from KEGG and PGD. Reactions supported by sufficient evidence and with specific enough functional annotations were incorporated into the model. For every accepted reaction its reversibility was assessed basing on assignments in KEGG pathways as well as information from BRENDA database. For reactions with inconsistent assignments a decision about reversibility was made basing on analysis of the reaction as well as its reversibility in other organisms. Hereby, a first version of the metabolic model was created (iJP815^pre1^).

The next step involved assessing whether the reconstructed metabolic network is able to produce energy from glucose. This was achieved by running FBA with ATP production set as the objective function. Subsequently, the ability of the model to grow *in silico* on glucose was tested. Successful *in silico* growth indicates that every chemical compound belonging to the biomass equation can be synthesized from present sources, using the reactions contained in the model. Since the exact cellular composition of *P. putida* is not known, the composition of *E. coli* biomass was used as an approximation. This test was performed by running FBA with production of each biomass constituent set as the objective. If a compound could not be synthesized, the gaps in the pathway leading to it were identified manually and a search was performed for reactions that could fill the gaps. If this approach was unsuccessful, gaps were filled with reactions from the *E. coli* model. This yielded the second version of the reconstruction (iJP815^pre2^).

The third round of reconstruction consisted of two sub-steps. First, the compounds for which transport proteins exist were identified and appropriate reactions added. Second, the results of BIOLOG carbon-source utilization experiments were compared with *in silico* simulations for growth on those compounds. It was assumed that the ability to grow *in silico* on the particular compound as the sole carbon source approximates the *in vivo* utilization. For those compounds that did not show *in silico* growth, a literature search was performed in order to identify possible pathways of utilization. The results of this search, in the form of reactions and GPRs, were added to the model. The outcome was the final version of the model (iJP815).

### Comparison of Growth Yields with the Continuous Culture Experiments

Growth yields on sources of basic elements (C,N,P,S) were compared with experimental values obtained by Duetz et al. [37]. The yields of the model were computed using FBA, by setting the growth rate to the value of the dilution rate used in experiments and subsequently minimizing for consumption of source of respective element (succinate, ammonia, phosphate and sulfate).

### Computational Methods

The model was created and maintained using ToBiN (Toolbox for Biochemical Networks, http://www.lifewizz.com). The optimizations (FBA, FVA, OptKnock) were computed by free, open source, solvers from the COIN-OR family (COmputational INfrastructure for Operations Research, http://www.coin-or.org) or by the lp_solve ver. 5.5 (http://lpsolve.sourceforge.net/5.5/) software package. All computations were performed on a Personal Computer with a Intel Core 2 2.40 GHz CPU and 2GB of RAM.

### Experimental Methods

#### Media and chemicals

*P. putida* KT2440 was grown either on EM-medium (Bacto Trypton – 20 g, Yeast-Extract – 5 g, NaCl – 5 g, Glucose 0.5%, H_2_O_dist_ at 1000 ml; the glucose was as 10% solution autoclaved separately and added in appropriate amount) or SOC-medium (Bacto Trypton – 2%, Yeast-extract – 0.5%, Glucose – 20 nM, NaCl – 10 mM, KCl – 2.5 mM, MgCl_2_ – 10 mM, MgSO_4_ – 10 mM, H_2_O_dist_ ad 1000 ml; magnesium salts were autoclaved separately and subsequently merged with the remaining components) or minimal medium (10×; Na_2_HPO_4_ – 50 g, KH_2_PO_4_ – 100 g, MgSO_4_×7H_2_O – 2 g, (NH_4_)_2_SO_4_ – 20 g, CaCl_2_ – 0.01 g, FeSO_4_×7H2O – 0.01 g, H_2_O_dist_ ad 1000 ml; the potassium and sodium salts were dissolved separately and subsequently mixed with other dissolved salts; pH was set to 7.0 by adding 10 mM NaOH) with different compounds as the sole carbon source.

#### BIOLOG substrate utilization experiments

P*seudomonas putida* KT2440 was tested for its ability to utilize various carbon sources using BIOLOG GN2 Microplates [31] (BIOLOG Inc. Hayward, CA, USA). All procedures were performed as indicated by the manufacturer. Bacteria were grown overnight in 28°C on a Biolog Universal Growth agar plate. Afterwards they were swabbed from the surface of the plate and suspended in GN inoculating fluid. Each well of the Microplate was inoculated with 150 µl of bacterial suspension and the plate was incubated in 28°C for 24 h. Subsequently the plate was read by a microplate reader and the read-outs were analyzed with MicroLog3 4.20 software.

#### Growth experiments

If not stated differently, cells were grown on agar plates overnight in 30°C.

#### Transposon mutagenesis

The mutants of *P. putida* were created using an *in vitro* transposition system (Epicentre Technologies, Madison, Wisconsin, USA) [84]. This system bases on a hyper-reactive Tn*5*-transposase and Tn5-Transposome that, in the absence of magnesium ions, builds a stable synaptic complex, which can be transmitted into the cell via electroporation. To render *Pseudomonas putida* KT2440 electrocompetent, cells were grown in 50 ml of EM-medium to OD_600_ of 0.6 to 1.0 and subsequently cooled on ice for 15 minutes. The cells were centrifuged (4000 g, 4°C) and washed twice with H_2_O_dist_. The cells were washed twice in 0.3 M cold solution of sucrose and resuspended in 0.5 to 1.0 ml of 0.3 M sucrose solution. The electrocompetent cell were used for transformation by electroporation with Gene Pulser (BioRad, Munich, Germany) using the EZ:TN <Kan-2> Tnp Transposome. 20–40 µl of cells was mixed with 1–2 µl of DNA in ice-cooled cuvette. The electroporation setting were 25 µF, 200 Ω, and 1.7 or 2.5 for the gap size 0.1 and 0.2 cm, respectively. After two hours of incubation in SOC-medium, transformants were selected on EM agar plates with 60 µg/ml of kanamycin. Selection of auxotrophic mutants was performed on minimal medium with acetate as the sole carbon source, by replica-plating *P. putida* KT2440::Tn5(Kan^r^) strains on the minimal and EM media.

#### Identification of flanking sequences

The auxotrophic *P. putida* KT2440::Tn5(Kan^r^) mutants were genotyped by enrichment of either flanking sequences of transposon insertions using PCR [85],[86]. Two rounds of amplifications were performed using primers specific to the ends of transposons and random primers that can anneal to the chromosome. In the first round of amplification the Kan-2 RP1 (5′-GCAATGTAACATCAGAGATTTTGAG-3′) primer complementary to the end of Tn5-element and the arbitrary primer ARB1 (5′-GGCCACGCGTCGACTAGTACNNNNNNNNNNGATAT-3′) were used. A 1 µl of supernatant from a *P. putida* KT2440 lysate was used as the DNA-template. The PCR-reaction was performed in following mixture [H_2_O_dist_ – 28.7 µl, incubation buffer(10×) – 5 µl, dNTPs(5 µM) – 5 µl, primer(10 µM) – 2,5 µl, Taq DNA-polymerase (5U/µl) – 0.2 µl] under following conditions: (i) 5 m at 95°C, (ii) 30×[30 s at 30°C, 90 s at 72°C], (iii) 30×[30 s at 95°C, 30 s at 45°C 120 s at 72°C]. In the second round of amplification a 5 µl of product of the first PCR-reaction was used as the DNA-template, together with the primers TnINT Rev (5′-GAGACACAATTCATCGATGGTTAGTG-3′) and ARB-2 (5′-GGCCACGCGTCGACTAGTAC-3′). The reaction conditions were following: 30×[30 s at 95°C, 30 s at 45°C, 120 s at 72°C]. The PCR-products were purified with “QIAquick- spin PCR Purification Kit” (Qiagen GmbH, Hilden, Germany) according to manufacturer's instructions. Subsequently, the sequencing procedure was performed. 200–500 µg of dsDNA in normal sequencing vectors (pBlueskript, pUC18, etc.) with 10 pmol of primer (TnINT Rev) and 6 µl of “Big Dye Terminator v. 2.0 Ready Reaction Mix” were mixed in total volume of 10 µl. The conditions of the reaction were following: 25×[30 s at 95°C, 30 s at 60°C, 4 m at 60°C]. After the cycle sequencing the remaining dNTP were removed using “Dye Ex Spin Kit” (Qiagen GmbH, Hilden, Germany) according to manufacturer's instructions. To the purified product 50 µl sterile MiliQ-H_2_O was added and the DNA was precipitated wit 250 µl Ethanol (100% v/v) for 30 min at 16000×g in the room temperature. The supernatant was removed and the pellet washed with 250 µl of ethanol (100% v/v), precipitated again by centrifugation (16000×g, RT, 10 min) and dried in vacuum-centrifuge. All the DNA-pellets were stored in −20°C in 20 µl Hi-Di Formamide (PE Biosystems) until sequencing. The sequencing was performed with ABI PRISM 377 sequencer [87]. The fluorescence signals were analyzed with ABI PRISM 3100 Genetic Analyser and the obtained sequences compared with *P. putida* KT2440 genome sequence.

## Supporting Information

Figure S1 Influence of biomass composition on the growth yield. Each bar represents a biomass with the fraction of one compound modified.
(0.18 MB TIF)

Figure S2 Influence of maintenance values on the growth yield. (A) influence when the glucose is supplied with the rate of 2.2 mmol⋅g_DW_^−1^⋅h^−1^ (B) influence when the glucose is supplied with rate 10 mmol⋅g_DW_^−1^⋅h^−1^.
(1.07 MB TIF)

Figure S3 Predictions of the fluxes in the central metabolism when the network structure assumed by the authors of the ^13^C measurements is used.
(1.97 MB TIF)

Figure S4 Influence of biomass composition on the prediction of internal fluxes. (A) results obtained from Optimal FVA (B) results obtained from suboptimal FVA.
(0.63 MB TIF)

Figure S5 Analysis of variability of particular reactions. Comparison of sizes of particular variability groups in various conditions.
(0.29 MB TIF)

Figure S6 Flux Coupling Finder, comparison of numbers of coupled reaction sets with respect to their size.
(0.67 MB TIF)

Table S1 Comparison of metabolic reconstruction created up to date
(0.07 MB DOC)

Table S2 BIOLOG assay details
(0.02 MB XLS)

Table S3 Distribution of variable reactions among pathways
(0.05 MB DOC)

Table S4 *In silico* growth results of the mutant strains
(0.05 MB DOC)

Table S5 Assembly of various properties of the reactions belonging to iJP815
(0.36 MB XLS)

Text S1 Supplementary methods
(0.11 MB DOC)

Text S2 Supplementary results
(0.05 MB DOC)

Text S3 Publications that contributed to the iJP815 reconstruction process
(0.02 MB DOC)

## References

[1] Timmis KN (2002). *Pseudomonas putida*: a cosmopolitan opportunist
par excellence.. Environ Microbiol.

[2] dos Santos VAPM, Heim S, Moore ERB, Stratz M, Timmis KN (2004). Insights into the genomic basis of niche specificity of
*Pseudomonas putida* KT2440.. Environ Microbiol.

[3] Moore ERB, Tindall BJ, Martins dos Santos VAP, Pieper DH, Ramos JL, Dworkin M, Falkow S, Rosenberg E, Schleifer K, Stackebrandt E (2006). Nonmedical: *Pseudomonas*.. The Prokaryotes: A Handbook on the Biology of Bacteria..

[4] Mosqueda G, Ramos-Gonzalez MI, Ramos JL (1999). Toluene metabolism by the solvent-tolerant *Pseudomonas
putida* DOT-T1 strain, and its role in solvent
impermeabilization.. Gene.

[5] de Bont JAM (1998). Solvent-tolerant bacteria in biocatalysis.. Trends Biotechnol.

[6] Wierckx NJP, Ballerstedt H, de Bont JAM, Wery J (2005). Engineering of solvent-tolerant *Pseudomonas
putida* S12 for bioproduction of phenol from glucose.. Appl Environ Microbiol.

[7] Nijkamp K, van Luijk N, de Bont JAM, Wery J (2005). The solvent-tolerant *Pseudomonas putida* S12 as
host for the production of cinnamic acid from glucose.. Appl Microbiol Biotechnol.

[8] Choi WJ, Lee EY, Cho MH, Choi CY (1997). Enhanced production of cis,cis-muconate in a cell-recycle
bioreactor.. J Ferment Bioeng.

[9] Ramos-Gonzalez MI, Ben-Bassat A, Campos MJ, Ramos JL (2003). Genetic engineering of a highly solvent-tolerant
*Pseudomonas putida* strain for biotransformation of
toluene to p-hydroxybenzoate.. Appl Environ Microbiol.

[10] Verhoef S, Ruijssenaars HJ, de Bont JAM, Wery J (2007). Bioproduction of p-hydroxybenzoate from renewable feedstock by
solvent-tolerant *Pseudomonas putida* S12.. J Biotechnol.

[11] Nijkamp K, Westerhof RGM, Ballerstedt H, de Bont JAM, Wery J (2007). Optimization of the solvent-tolerant *Pseudomonas
putida* S12 as host for the production of p-coumarate from glucose.. Appl Microbiol Biotechnol.

[12] Stephan S, Heinzle E, Wenzel SC, Krug D, Muller R (2006). Metabolic physiology of *Pseudomonas putida* for
heterologous production of myxochromide.. Process Biochem.

[13] Schmid A, Dordick JS, Hauer B, Kiener A, Wubbolts M (2001). Industrial biocatalysis today and tomorrow.. Nature.

[14] Nelson KE, Weinel C, Paulsen IT, Dodson RJ, Hilbert H (2002). Complete genome sequence and comparative analysis of the
metabolically versatile *Pseudomonas putida* KT2440.. Environ Microbiol.

[15] Wackett LP (2003). *Pseudomonas putida*—a versatile
biocatalyst.. Nat Biotechnol.

[16] Jimenez JI, Minambres B, Garcia JL, Diaz E (2002). Genomic analysis of the aromatic catabolic pathways from
*Pseudomonas putida* KT2440.. Environ Microbiol.

[17] Huijberts GNM, Eggink G (1996). Production of poly(3-hydroxyalkanoates) by *Pseudomonas
putida* KT2442 in continuous cultures.. Appl Microbiol Biotechnol.

[18] Steinbüchel A, Hein S (2001). Biochemical and molecular basis of microbial synthesis of
polyhydroxyalkanoates in microorganisms.. Adv Biochem Eng Biotechnol.

[19] Price ND, Reed JL, Palsson BO (2004). Genome-scale models of microbial cells: evaluating the
consequences of constraints.. Nat Rev Microbiol.

[20] Reed JL, Palsson BO (2003). Thirteen years of building constraint-based *in
silico* models of *Escherichia coli*.. J Bacteriol.

[21] Papin JA, Price ND, Wiback SJ, Fell DA, Palsson BO (2003). Metabolic pathways in the post-genome era.. Trends Biochem Sci.

[22] Varma A, Palsson BO (1994). Stoichiometric flux balance models quantitatively predict growth
and metabolic by-product secretion in wild-type *Escherichia
coli* W3110.. Appl Environ Microbiol.

[23] Covert MW, Knight EM, Reed JL, Herrgard MJ, Palsson BO (2004). Integrating high-throughput and computational data elucidates
bacterial networks.. Nature.

[24] Price ND, Papin JA, Schilling CH, Palsson BO (2003). Genome-scale microbial *in silico* models: the
constraints-based approach.. Trends Biotechnol.

[25] Joyce AR, Palsson BO (2007). Toward whole cell modeling and simulation: comprehensive
functional genomics through the constraint-based approach.. Prog Drug Res.

[26] Lee KH, Park JH, Kim TY, Kim HU, Lee SY (2007). Systems metabolic engineering of *Escherichia
coli* for l-threonine production.. Mol Syst Biol.

[27] Pharkya P, Burgard AP, Maranas CD (2004). OptStrain: a computational framework for redesign of microbial
production systems.. Genome Res.

[28] Burgard AP, Pharkya P, Maranas CD (2003). OptKnock: a bilevel programming framework for identifying gene
knockout strategies for microbial strain optimization.. Biotechnol Bioeng.

[29] Pharkya P, Burgard AP, Maranas CD (2003). Exploring the overproduction of amino acids using the bilevel
optimization framework OptKnock.. Biotechnol Bioeng.

[30] Papin JA, Stelling J, Price ND, Klamt S, Schuster S (2004). Comparison of network-based pathway analysis methods.. Trends Biotechnol.

[31] Bochner BR, Gadzinski P, Panomitros E (2001). Phenotype microarrays for high-throughput phenotypic testing and
assay of gene function.. Genome Res.

[32] Fischer E, Zamboni N, Sauer U (2004). High-throughput metabolic flux analysis based on gas
chromatography–mass spectrometry derived ^13^C
constraints.. Anal Biochem.

[33] Reed JL, Vo TD, Schilling CH, Palsson BO (2003). An expanded genome-scale model of *Escherichia
coli* K-12 (iJR904 GSM/GPR).. Genome Biol.

[34] Osterman A, Overbeek R (2003). Missing genes in metabolic pathways: a comparative genomics
approach.. Curr Opin Chem Biol.

[35] Kanehisa M, Goto S (2000). KEGG: Kyoto Encyclopedia of Genes and Genomes.. Nucleic Acids Res.

[36] Revelles O, Espinosa-Urgel M, Fuhrer T, Sauer U, Ramos JL (2005). Multiple and interconnected pathways for l-lysine
catabolism in *Pseudomonas putida* KT2440.. J Bacteriol.

[37] Duetz WA, Marques S, Wind B, Ramos JL, van Andel JG (1996). Catabolite repression of the toluene degradation pathway in
*Pseudomonas putida* harboring pWWO under various
conditions of nutrient limitation in chemostat culture.. Appl Environ Microbiol.

[38] Pfeiffer T, Schuster S (2005). Game-theoretical approaches to studying the evolution of
biochemical systems.. Trends Biochem Sci.

[39] Schuster S, Pfeiffer T, Fell DA (2008). Is maximization of molar yield in metabolic networks favoured by
evolution?. J Theor Biol.

[40] Pramanik J, Keasling JD (1998). Effect of *Escherichia coli* biomass composition
on central metabolic fluxes predicted by a stoichiometric model.. Biotechnol Bioeng.

[41] Mahadevan R, Schilling CH (2003). The effects of alternate optimal solutions in constraint-based
genome-scale metabolic models.. Metab Eng.

[42] Russell JB, Cook GM (1995). Energetics of bacterial growth: balance of anabolic and catabolic
reactions.. Microbiol Rev.

[43] Hempfling WP, Mainzer SE (1975). Effects of varying carbon source limiting growth on yield and
maintenance characteristics of *Escherichia coli* in
continuous culture.. J Bacteriol.

[44] Mainzer SE, Hempfling WP (1976). Effects of growth temperature on yield and maintenance during
glucose-limited continuous culture of *Escherichia coli*.. J Bacteriol.

[45] Isken S, Derks A, Wolffs PFG, de Bont JAM (1999). Effect of organic solvents on the yield of solvent-tolerant
*Pseudomonas putida* S12.. Appl Environ Microbiol.

[46] Fieschko J, Humphrey AE (1984). Statistical analysis in the estimation of maintenance and true
growth yield coefficients.. Biotechnol Bioeng.

[47] Bratbak G (1985). Bacterial biovolume and biomass estimations.. Appl Environ Microbiol.

[48] Burgard AP, Nikolaev EV, Schilling CH, Maranas CD (2004). Flux coupling analysis of genome-scale metabolic network
reconstructions.. Genome Res.

[49] Fuhrer T, Fischer E, Sauer U (2005). Experimental identification and quantification of glucose
metabolism in seven bacterial species.. J Bacteriol.

[50] del Castillo T, Ramos JL, Rodriguez-Herva JJ, Fuhrer T, Sauer U (2007). Convergent peripheral pathways catalyze initial glucose
catabolism in *Pseudomonas putida*: genomic and flux
analysis.. J Bacteriol.

[51] Cozzone AJ (1998). Regulation of acetate metabolism by protein phosphorylation in
enteric bacteria.. Annu Rev Microbiol.

[52] Teusink B, Wiersma A, Molenaar D, Francke C, de Vos WM (2006). Analysis of growth of *Lactobacillus plantarum*
WCFS1 on a complex medium using a genome-scale metabolic model.. J Biol Chem.

[53] Schuetz R, Kuepfer L, Sauer U (2007). Systematic evaluation of objective functions for predicting
intracellular fluxes in *Escherichia coli*.. Mol Syst Biol.

[54] Pal C, Papp B, Lercher MJ, Csermely P, Oliver SG (2006). Chance and necessity in the evolution of minimal metabolic
networks.. Nature.

[55] Jensen PR, Michelsen O (1992). Carbon and energy metabolism of atp mutants of
*Escherichia coli*.. J Bacteriol.

[56] von Meyenburg K, Jorgensen BB, Nielsen J, Hansen FG (1982). Promoters of the atp operon coding for the membrane-bound ATP
synthase of *Escherichia coli* mapped by Tn10 insertion
mutations.. Mol Gen Genet.

[57] Kornberg HL (1966). Role and control of glyoxylate cycle in *Escherichia
coli*.. Biochem J.

[58] Fischer E, Sauer U (2005). Large-scale in vivo flux analysis shows rigidity and suboptimal
performance of *Bacillus subtilis* metabolism.. Nat Genet.

[59] Oh YK, Palsson BO, Park SM, Schilling CH, Mahadevan R (2007). Genome-scale reconstruction of metabolic network in
*Bacillus subtilis* based on high-throughput phenotyping
and gene essentiality data.. J Biol Chem.

[60] Oberhardt MA, Puchalka J, Fryer KE, dos Santos VAPM, Papin JA (2008). Genome-scale metabolic network analysis of the opportunistic
pathogen *Pseudomonas aeruginosa* PAO1.. J Bacteriol.

[61] Steinbuchel A (2001). Perspectives for biotechnological production and utilization of
biopolymers: metabolic engineering of polyhydroxyalkanoate biosynthesis
pathways as a successful example.. Macromol Biosci.

[62] Giavaresi G, Tschon M, Borsari V, Daly JH, Liggat JJ (2004). New polymers for drug delivery systems in orthopaedics: in vivo
biocompatibility evaluation.. Biomed Pharmacother.

[63] van der Walle GAM, de Koning GJM, Weusthuis RA, Eggink G (2001). Properties, modifications and applications of biopolyesters.. Adv Biochem Eng Biotechnol.

[64] Klinke S, Dauner M, Scott G, Kessler B, Witholt B (2000). Inactivation of isocitrate lyase leads to increased production of
medium-chain-length poly(3-hydroxyalkanoates) in *Pseudomonas
putida*.. Appl Environ Microbiol.

[65] Patil KR, Akesson M, Nielsen J (2004). Use of genome-scale microbial models for metabolic engineering.. Curr Opin Biotechnol.

[66] Jamshidi N, Palsson BO (2008). Formulating genome-scale kinetic models in the post-genome era.. Mol Syst Biol.

[67] Joyce AR, Palsson BO (2006). The model organism as a system: integrating
‘omics’ data sets.. Nat Rev Mol Cell Biol.

[68] Seker S, Beyenal H, Salih B, Tanyolac A (1997). Multi-substrate growth kinetics of *Pseudomonas
putida* for phenol removal.. Appl Microbiol Biotechnol.

[69] Kumar A, Kumar S, Kumar S (2005). Biodegradation kinetics of phenol and catechol using
*Pseudomonas putida* MTCC 1194.. Biochem Eng J.

[70] Wang SJ, Loh KC (2001). Biotransformation kinetics of *Pseudomonas putida*
for cometabolism of phenol and 4-chlorophenol in the presence of sodium
glutamate.. Biodegradation.

[71] Abuhamed T, Bayraktar E, Mehmetoglu T, Mehmetoglu U (2004). Kinetics model for growth of *Pseudomonas putida*
F1 during benzene, toluene and phenol biodegradation.. Process Biochem.

[72] Edwards JS, Palsson BO (2000). Metabolic flux balance analysis and the *in
silico* analysis of *Escherichia coli* K-12 gene
deletions.. BMC Bioinformatics.

[73] Vanrolleghem PA, Heijnen JJ (1998). A structured approach for selection among candidate metabolic
network models and estimation of unknown stoichiometric coefficients.. Biotechnol Bioeng.

[74] Palsson BO (2006). Systems Biology: Properties of Reconstructed Networks..

[75] Varma A, Palsson BO (1993). Metabolic capabilities of Escherichia-Coli .1. Synthesis of
biosynthetic precursors and cofactors.. J Theor Biol.

[76] Lee JM, Gianchandani EP, Papin JA (2006). Flux balance analysis in the era of metabolomics.. Brief Bioinform.

[77] Edwards JS, Palsson BO (2000). The *Escherichia coli* MG1655 *in
silico* metabolic genotype: Its definition, characteristics, and
capabilities.. Proc Natl Acad Sci U S A.

[78] Schuster S, Fell DA, Dandekar T (2000). A general definition of metabolic pathways useful for systematic
organization and analysis of complex metabolic networks.. Nature Biotechnology.

[79] Reed JL, Palsson BO (2004). Genome-scale *in silico* models of E-coli have
multiple equivalent phenotypic states: Assessment of correlated reaction
subsets that comprise network states.. Genome Res.

[80] Bonarius HPJ, Schmid G, Tramper J (1997). Flux analysis of underdetermined metabolic networks: the quest
for the missing constraints.. Trends Biotechnol.

[81] Kanehisa M, Goto S, Hattori M, Aoki-Kinoshita KF, Itoh M (2006). From genomics to chemical genomics: new developments in KEGG.. Nucleic Acids Res.

[82] Winsor GL, Lo R, Sui SJH, Ung KSE, Huang SS (2005). *Pseudomonas aeruginosa* Genome Database and
PseudoCAP: facilitating community-based, continually updated, genome
annotation.. Nucleic Acids Res.

[83] Schomburg I, Chang A, Schomburg D (2002). BRENDA, enzyme data and metabolic information.. Nucleic Acids Res.

[84] Goryshin IY, Reznikoff WS (1998). Tn5 in vitro transposition.. J Biol Chem.

[85] Caetano-Anolles G (1993). Amplifying DNA with arbitrary oligonucleotide primers.. Genome Res.

[86] O'Toole GA, Kolter R (1998). Initiation of biofilm formation in *Pseudomonas
fluorescens* WCS365 proceeds via multiple, convergent signalling
pathways: a genetic analysis.. Mol Microbiol.

[87] Sanger F, Nicklen S, Coulson AR (1977). DNA sequencing with chain-terminating inhibitors.. Proc Natl Acad Sci U S A.

[88] Hoschle B, Gnau V, Jendrossek D (2005). Methylcrotonyl-CoA and geranyl-CoA carboxylases are involved in
leucine/isovalerate utilization (Liu) and acyclic terpene utilization (Atu),
and are encoded by liuB/liuD and atuC/atuF, in *Pseudomonas
aeruginosa*.. Microbiology.

