# Supplementary figures and images for: Genome-Scale Reconstruction and Analysis of the Pseudomonas putida KT2440 Metabolic Network Facilitates Applications in Biotechnology
Source: PLoS Comput Biol. 2008 Oct 31;4(10):e1000210. doi: 10.1371/journal.pcbi.1000210 (PMC2563689; doi:10.1371/journal.pcbi.1000210)

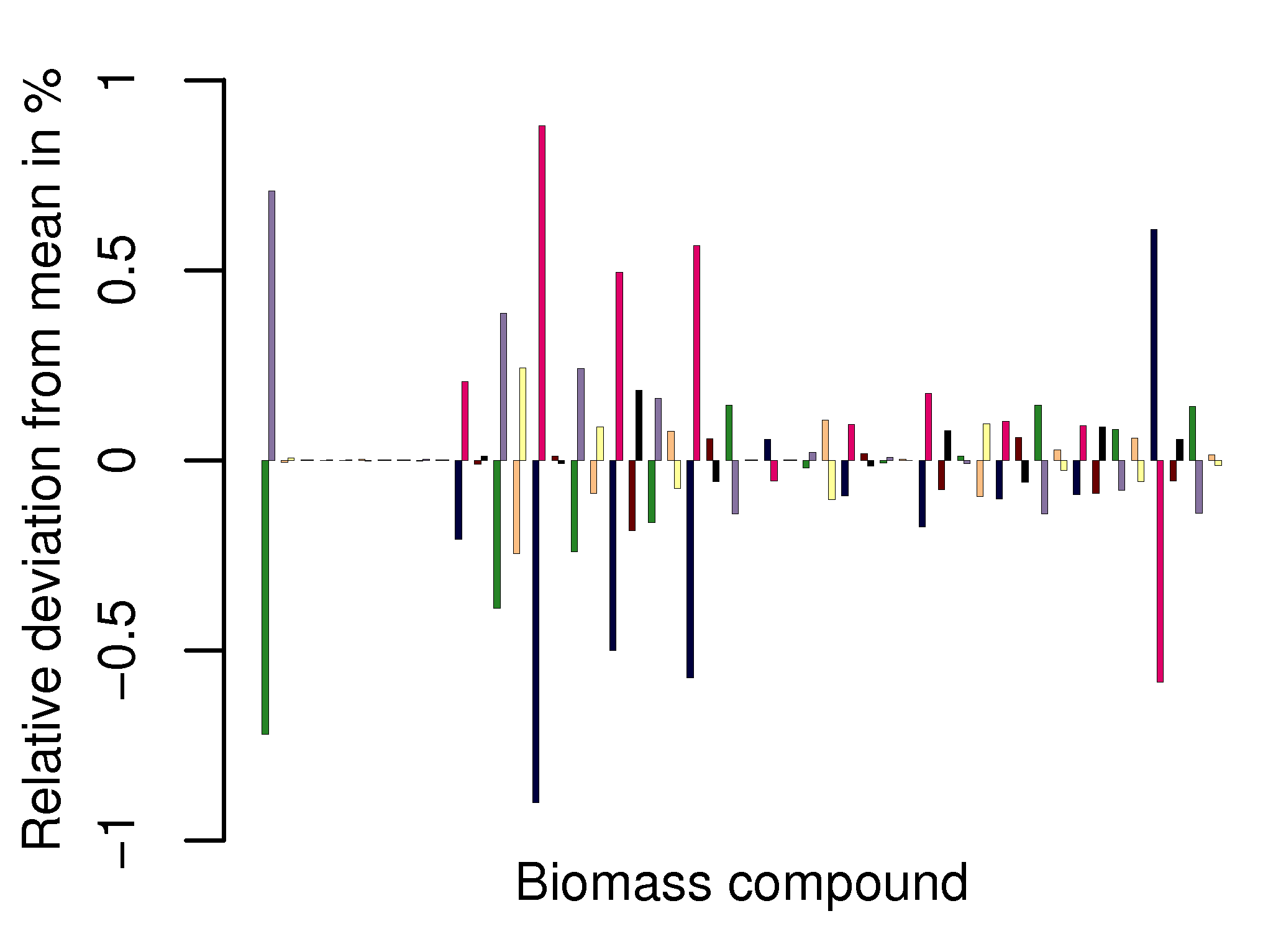

Supplement: Figure S1 — Influence of biomass composition on the growth yield. Each bar represents a biomass with the fraction of one compound modified. (0.18 MB TIF) [file pcbi.1000210.s001.tif]

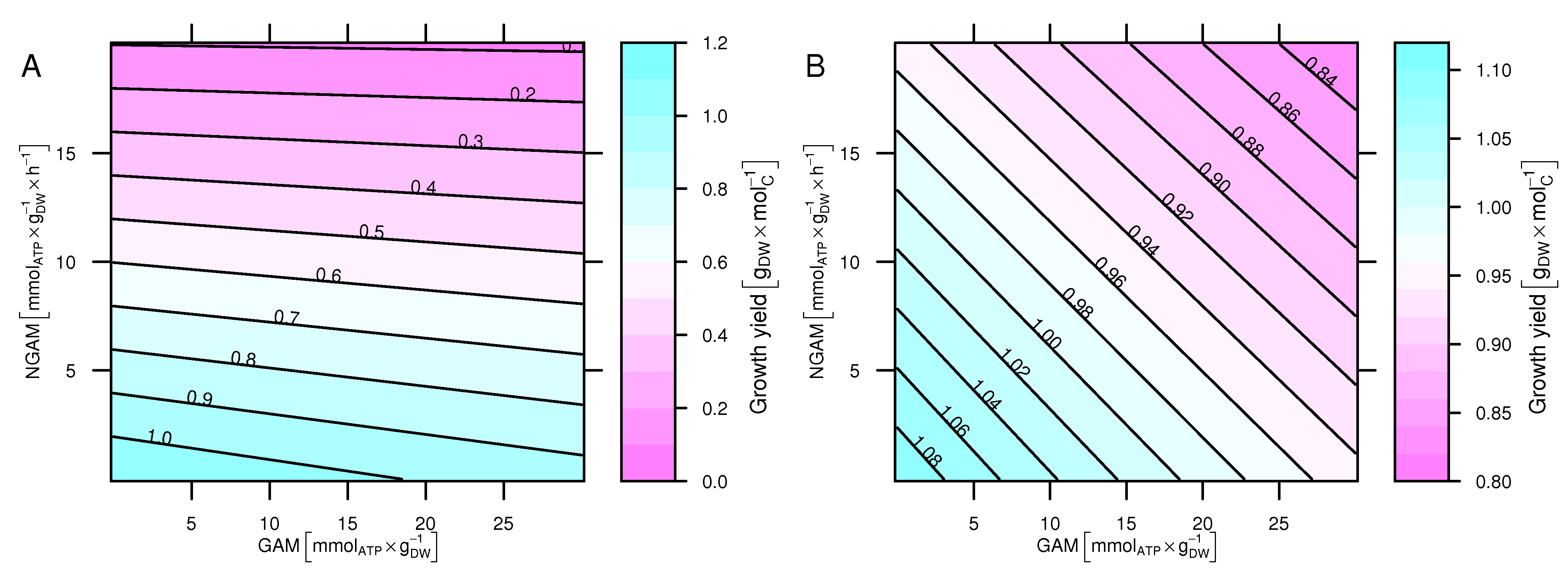

Supplement: Figure S2 — Influence of maintenance values on the growth yield. (A) influence when the glucose is supplied with the rate of 2.2 mmol⋅gDW −1⋅h−1 (B) influence when the glucose is supplied with rate 10 mmol⋅gDW −1⋅h−1. (1.07 MB TIF) [file pcbi.1000210.s002.tif]

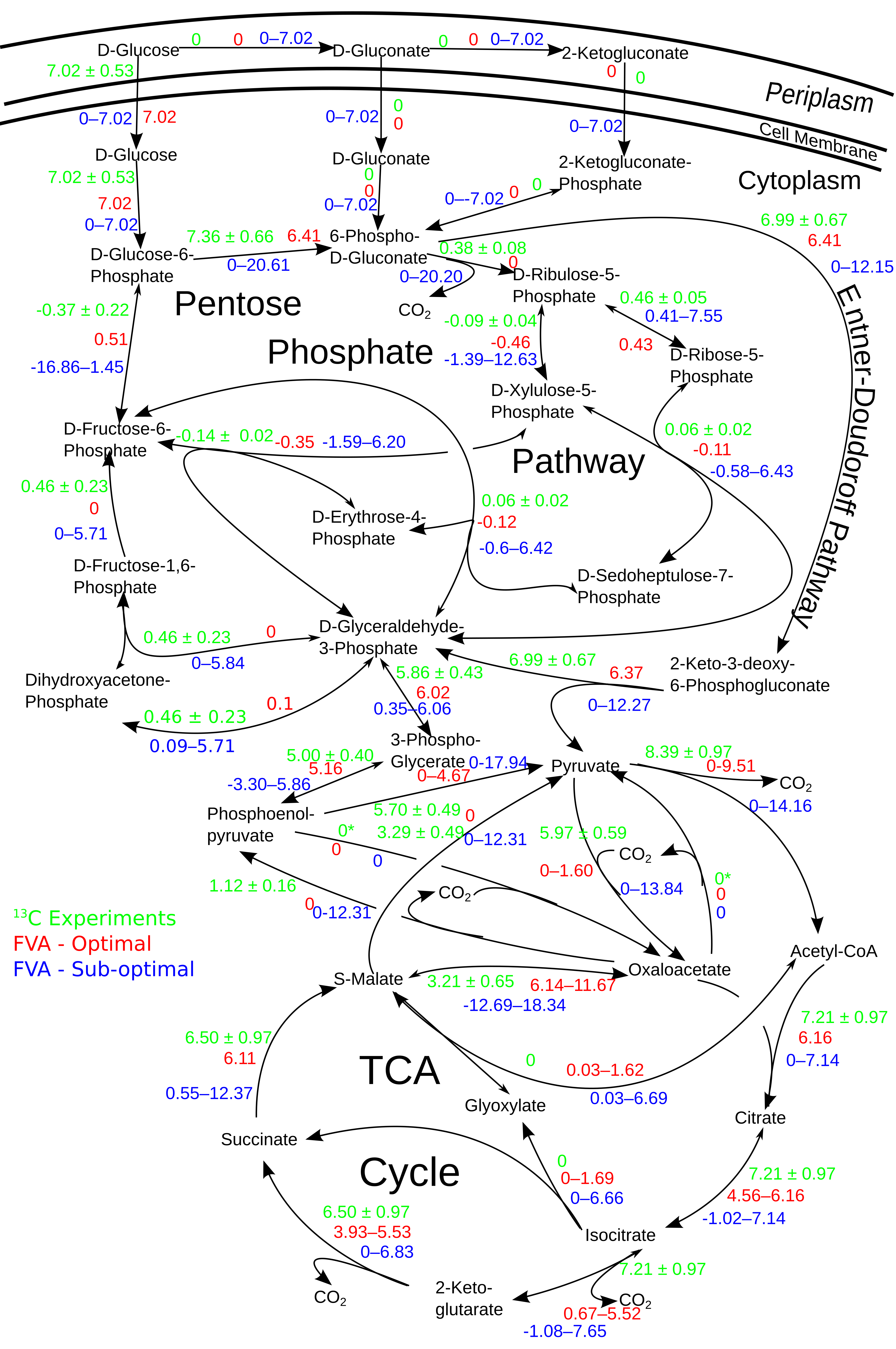

Supplement: Figure S3 — Predictions of the fluxes in the central metabolism when the network structure assumed by the authors of the 13C measurements is used. (1.97 MB TIF) [file pcbi.1000210.s003.tif]

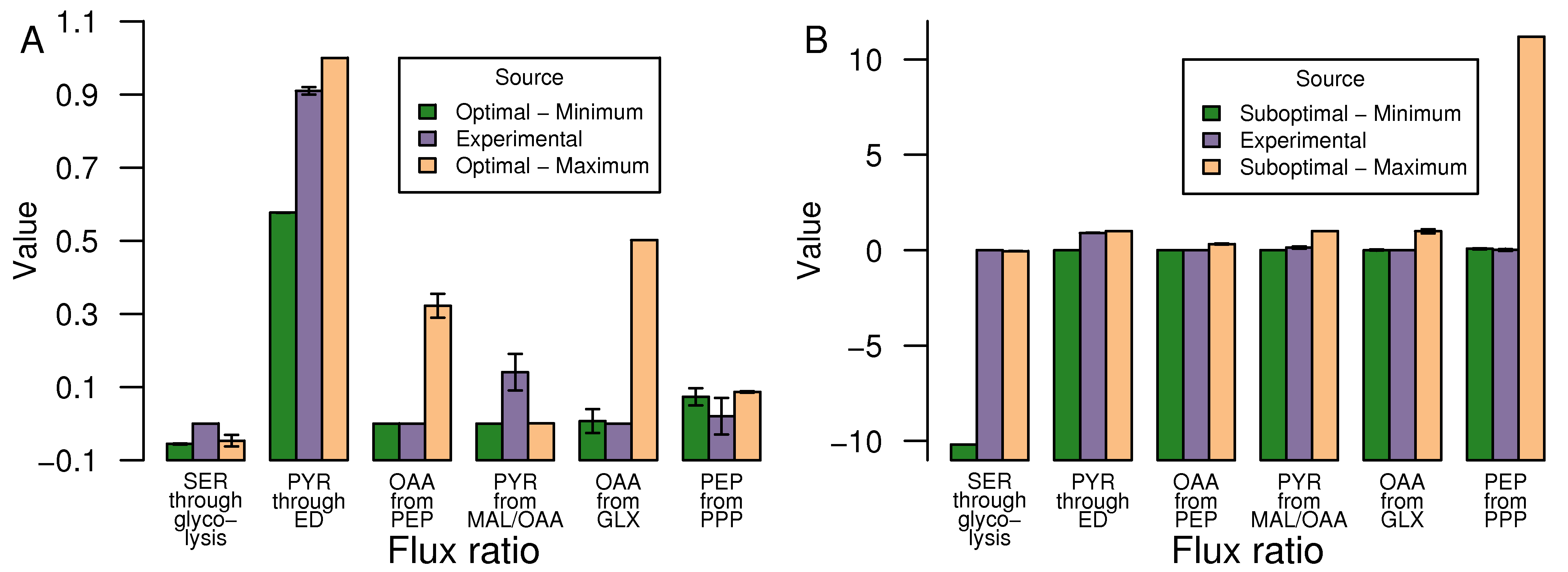

Supplement: Figure S4 — Influence of biomass composition on the prediction of internal fluxes. (A) results obtained from Optimal FVA (B) results obtained from suboptimal FVA. (0.63 MB TIF) [file pcbi.1000210.s004.tif]

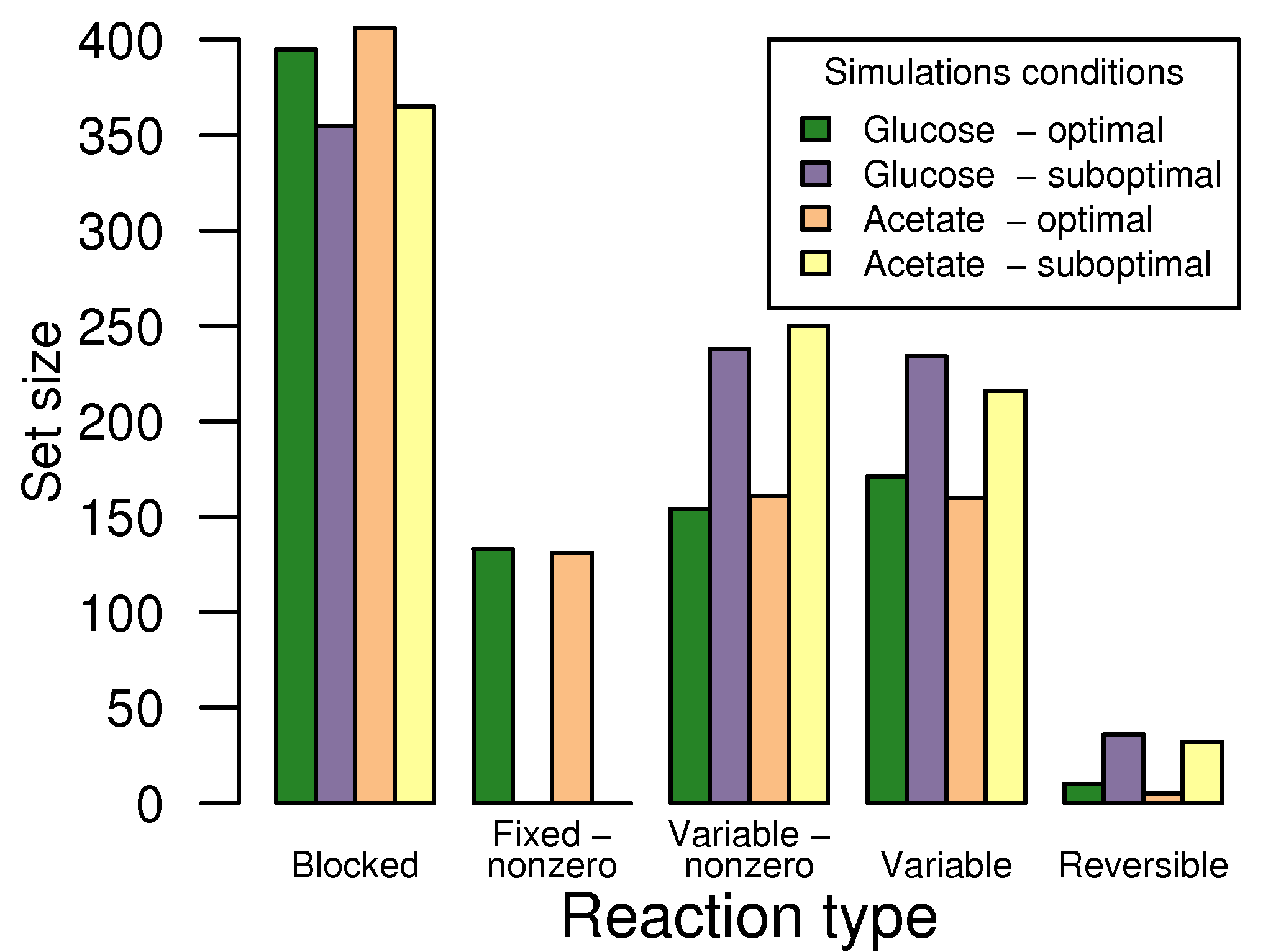

Supplement: Figure S5 — Analysis of variability of particular reactions. Comparison of sizes of particular variability groups in various conditions. (0.29 MB TIF) [file pcbi.1000210.s005.tif]

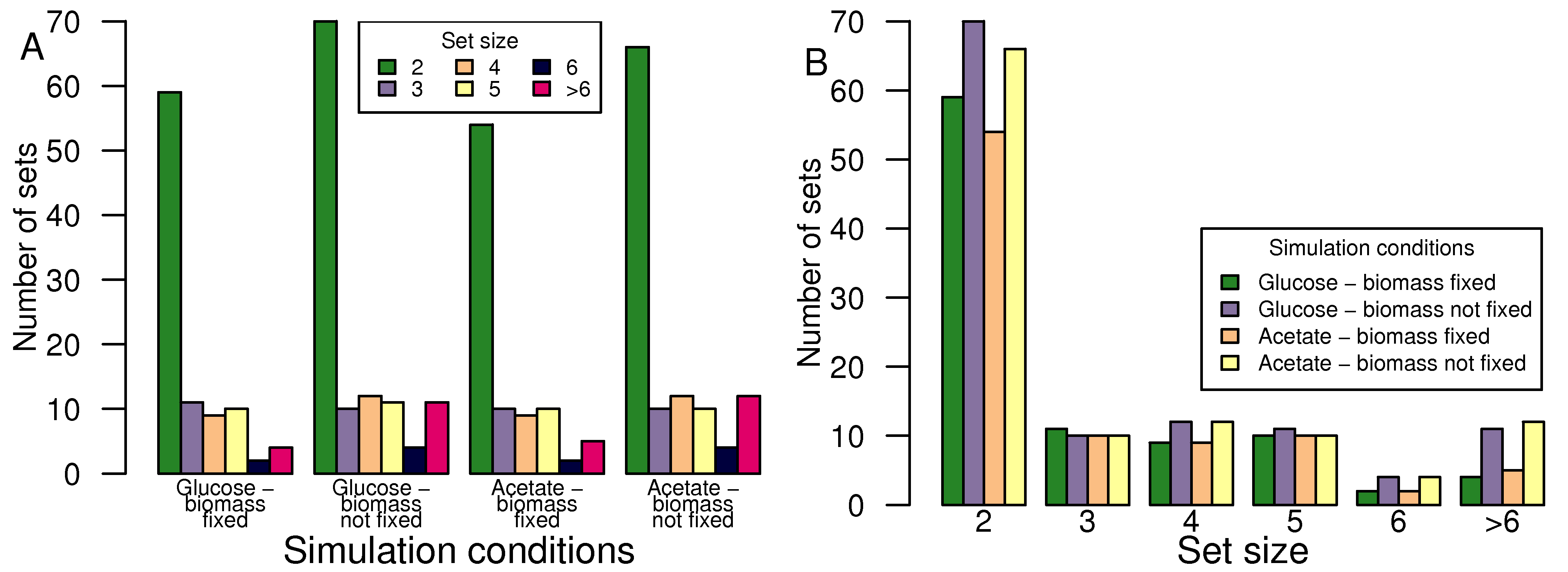

Supplement: Figure S6 — Flux Coupling Finder, comparison of numbers of coupled reaction sets with respect to their size. (0.67 MB TIF) [file pcbi.1000210.s006.tif]
