# Supplementary material for: Genome-Scale Reconstruction and Analysis of the Pseudomonas putida KT2440 Metabolic Network Facilitates Applications in Biotechnology
Source: PLoS Comput Biol. 2008 Oct 31;4(10):e1000210. doi: 10.1371/journal.pcbi.1000210 (PMC2563689; doi:10.1371/journal.pcbi.1000210)
Supplement: Table S1 — Comparison of metabolic reconstruction created up to date (0.07 MB DOC) [file pcbi.1000210.s007.doc]

**Table S1.** Comparison of metabolic reconstruction created up to date

| ***Organism*** | ***Genes*** | ***KEGG***  ***Metabolic Genes*** | ***Model***  ***Genes (% of KEGG)*** | ***Model Metabolites*** | ***Model Reactions*** | ***Reference*** |
| --- | --- | --- | --- | --- | --- | --- |
| ***Pseudomonas putida*** | **5446** | **1253** | **815 (65.0)** | **884** | **877** | **This work** |
| *Bacillus subtilis* | 4225 | 1008 | 614 (60.9) | 637 | 754 | [1] |
| *Escherichia coli* | 4405 | 1322 | 904 (68.3) | 625 | 931 | [2] |
| *Geobacter sulfurreducens* | 3530 | 692 | 588 (84.9) | 541 | 523 | [3] |
| *Haemophilus influenzae* | 1775 | 578 | 400 (69.2) | 451 | 461 | [4] |
| *Helicobacter pylori* | 1632 | 374 | 341 (91.1) | 485 | 476 | [5] |
| *Lactococcus lactis* | 2310 | 579 | 358 (61.8) | 422 | 621 | [6] |
| *Lactobacillus plantarum* | 3064 | 817 | 721 (88.2) | 531 | 643 | [7] |
| *Mannheimia succinici-producens* | 2463 | 711 | 335 (47.1) | 352 | 373 | [8] |
| *Mycobacterium tuberculosis* | 4039 | 988 | 726 (73.4) | 739 | 849 | [9] |
| *Pseudomonas aeruginosa* | 5649 | 1374 | 1056 (76.8) | 883 | 760 | [10] |
| *Staphylococcus aureus* | 2702 | 737 | 619 (83.9) | 571 | 641 | [11] |
| *Streptomyces coelicolor* | 8042 | 1210 | 700 (57.8) | 500 | 700 | [12] |

**REFERENCES**

1. Oh YK, Palsson BO, Park SM, Schilling CH, Mahadevan R (2007) Genome-scale reconstruction of metabolic network in Bacillus subtilis based on high-throughput phenotyping and gene essentiality data. Journal of Biological Chemistry 282: 28791-28799.

2. Reed JL, Vo TD, Schilling CH, Palsson BO (2003) An expanded genome-scale model of Escherichia coli K-12 (iJR904 GSM/GPR). Genome Biology 4: -.

3. Mahadevan R, Bond DR, Butler JE, Esteve-Nunez A, Coppi MV, et al. (2006) Characterization of metabolism in the Fe(III)-reducing organism Geobacter sulfurreducens by constraint-based modeling. Applied and Environmental Microbiology 72: 1558-1568.

4. Edwards JS, Palsson BO (1998) The Haemophilus influenzae metabolic genotype: Its definition and systems characteristics. Abstracts of Papers of the American Chemical Society 216: U268-U268.

5. Schilling CH, Covert MW, Famili I, Church GM, Edwards JS, et al. (2002) Genome-scale metabolic model of Helicobacter pylori 26695. Journal of Bacteriology 184: 4582-4593.

6. Oliveira AP, Nielsen J, Forster J (2005) Modeling Lactococcus lactis using a genome-scale flux model. Bmc Microbiology 5: -.

7. Teusink B, Wiersma A, Molenaar D, Francke C, de Vos WM, et al. (2006) Analysis of growth of Lactobacillus plantarum WCFS1 on a complex medium using a genome-scale metabolic model. Journal of Biological Chemistry 281: 40041-40048.

8. Hong SH, Kim JS, Lee SY, In YH, Choi SS, et al. (2004) The genome sequence of the capnophilic rumen bacterium Mannheimia succiniciproducens. Nature Biotechnology 22: 1275-1281.

9. Beste DJV, Hooper T, Stewart G, Bonde B, Avignone-Rossa C, et al. (2007) GSMN-TB: a web-based genome scale network model of Mycobacterium tuberculosis metabolism. Genome Biology 8: -.

10. Oberhardt MA, Puchalka J, Fryer KE, dos Santos VAPM, Papin JA (2008) Genome-scale metabolic network analysis of the opportunistic pathogen Pseudomonas aeruginosa PAO1. J Bacteriol: JB.01583-01507.

11. Becker SA, Palsson BO (2005) Genome-scale reconstruction of the metabolic network in Staphylococcus aureus N315: an initial draft to the two-dimensional annotation. Bmc Microbiology 5: -.

12. Borodina I, Krabben P, Nielsen J (2005) Genome-scale analysis of Streptomyces coelicolor A3(2) metabolism. Genome Research 15: 820-829.
