# Supplementary material for: Genome-Scale Reconstruction and Analysis of the Pseudomonas putida KT2440 Metabolic Network Facilitates Applications in Biotechnology
Source: PLoS Comput Biol. 2008 Oct 31;4(10):e1000210. doi: 10.1371/journal.pcbi.1000210 (PMC2563689; doi:10.1371/journal.pcbi.1000210)
Supplement: Table S3 — Distribution of variable reactions among pathways (0.05 MB DOC) [file pcbi.1000210.s009.doc]

**Table S3. Distribution of variable reactions among pathways.**

|  |  | ***Fraction of variable reactions (group 4 and 5)*** | | | | |
| --- | --- | --- | --- | --- | --- | --- |
| ***Pathway*** | ***Number of reactions*** | | ***Glucose*** | ***Glucose – sub-optimal*** | ***Acetate*** | ***Acetate – sub-optimal*** |
| Lysine degradation | 18 | | 0.89 | 1 | 0.89 | 1 |
| Valine, leucine and isoleucine degradation | 20 | | 0.85 | 0.9 | 0.85 | 0.9 |
| Propanoate metabolism | 10 | | 0.8 | 0.9 | 0.8 | 0.9 |
| Butanoate metabolism | 12 | | 0.58 | 0.92 | 0.58 | 0.83 |
| Arginine and proline metabolism | 20 | | 0.55 | 0.7 | 0.55 | 0.7 |
| Purine metabolism | 44 | | 0.55 | 0.57 | 0.48 | 0.57 |
| Citrate cycle (TCA cycle) | 11 | | 0.55 | 0.91 | 0.45 | 0.55 |
| Pyruvate metabolism | 17 | | 0.53 | 0.71 | 0.47 | 0.59 |
| Glutamate metabolism | 17 | | 0.47 | 0.53 | 0.47 | 0.53 |
| Folate biosynthesis | 15 | | 0.47 | 0.53 | 0.47 | 0.53 |
| Glycolysis / Gluconeogenesis | 11 | | 0.45 | 0.64 | 0.45 | 0.45 |
| Histidine metabolism | 13 | | 0.38 | 0.38 | 0.38 | 0.38 |
| One carbon pool by folate | 11 | | 0.36 | 0.45 | 0.36 | 0.45 |
| Glyoxylate and dicarboxylate metabolism | 14 | | 0.36 | 0.79 | 0.36 | 0.43 |
| Pentose phosphate pathway | 12 | | 0.33 | 0.83 | 0.5 | 0.8 |
| Alanine and aspartate metabolism | 16 | | 0.31 | 0.38 | 0.31 | 0.38 |
| Phenylalanine, tyrosine and tryptophan biosynthesis | 21 | | 0.29 | 0.38 | 0.29 | 0.38 |
| Carbon fixation | 14 | | 0.29 | 0.57 | 0.21 | 0.43 |
| Pyrimidine metabolism | 24 | | 0.21 | 0.29 | 0.21 | 0.29 |
| Glycine, serine and threonine metabolism | 14 | | 0.14 | 0.43 | 0.14 | 0.43 |
| Urea cycle and metabolism of amino groups | 16 | | 0.13 | 0.44 | 0.13 | 0.44 |
| Lysine biosynthesis | 13 | | 0.08 | 0.08 | 0.08 | 0.08 |
| Valine, leucine and isoleucine biosynthesis | 14 | | 0 | 0 | 0 | 0 |
| Lipopolysaccharide biosynthesis | 15 | | 0 | 0 | 0 | 0 |
