# Supplementary material for: Genome-Scale Reconstruction and Analysis of the Pseudomonas putida KT2440 Metabolic Network Facilitates Applications in Biotechnology
Source: PLoS Comput Biol. 2008 Oct 31;4(10):e1000210. doi: 10.1371/journal.pcbi.1000210 (PMC2563689; doi:10.1371/journal.pcbi.1000210)
Supplement: Table S4 — In silico growth results of the mutant strains (0.05 MB DOC) [file pcbi.1000210.s010.doc]

**Table S4.** *In silico* growth results of the mutant strains.

| ***Gene/ Strain*** | ***In silico growth on acetate*** | ***Gene/ Strain*** | ***In silico growth on acetate*** | ***Gene/ Strain*** | ***In silico growth on acetate*** |
| --- | --- | --- | --- | --- | --- |
| PP0083 | no | PP1471 | yes | PP4700 | no |
| PP0184 | no | PP1612 | no | PP4715 | no |
| PP0289 | no | PP1665 | no | PP4723 | no |
| PP0290 | no | PP1768 | yes | PP4724 | no |
| PP0292 | no | PP1815 | no | PP4725 | no |
| PP0293 | no | PP2000 | no | PP4782 | yes |
| PP0417 | no | PP2371 | no | PP4823 | no |
| PP0787 | no | PP4188 | yes | PP4909 | yes |
| PP0897 | yes | PP4189 | yes | PP4998 | no |
| PP0965 | no | PP4191 | no | PP5097 | no |
| PP0966 | no | PP4192 | no | PP5128 | no |
| PP0967 | no | PP4193 | no | PP5155 | yes |
| PP1025 | no | PP4194 | no | PP5185 | yes |
| PP1037 | no | PP4678 | no | PP5289 | no |
| PP1086 | yes | PP4679 | yes | PP5291 | no |
| PP1088 | no | PP4680 | yes | PP5335 | no |
| PP1470 | yes | PP4699 | no | PP5336 | no |
