# Supplementary material for: Genome-Scale Reconstruction and Analysis of the Pseudomonas putida KT2440 Metabolic Network Facilitates Applications in Biotechnology
Source: PLoS Comput Biol. 2008 Oct 31;4(10):e1000210. doi: 10.1371/journal.pcbi.1000210 (PMC2563689; doi:10.1371/journal.pcbi.1000210)
Supplement: Text S1 — Supplementary methods (0.11 MB DOC) [file pcbi.1000210.s012.doc]

**SUPPLEMENTARY METHODS**

**Constraint based models – mathematical explanation:**

In modeling a metabolic network, the stoichiometries of all reactions can be summarized in form of a stoichiometry matrix (***S***-matrix) which eases further analysis. The rows of the ***S***-matrix represent chemical compounds whereas columns represent distinct reactions, and coefficients populating the columns of the matrix depict the stoichiometry of reactions in the network. The ***S***-matrix couples the rates (fluxes) of the reactions (***v***) with changes in concentration of metabolites (***C***) in following form:

$$\frac{dC}{dt}=S\cdot v$$

A Pseudo Steady State Assumption (PSSA) assumes that the concentrations of the metabolites stay constant over the time. This introduces the following constraint on the above equation:

$0\equiv\frac{dC}{dt}=S_{I}\cdot v$
where ***S****_I_* corresponds to stoichiometric sub-matrix containing rows pertaining compounds to which the PSSA should apply (internal compounds). Application of the PSSA limits the attainable flux space to an open polyhedral cone immersed inside n-dimensional space (where n is the number of reactions). This cone forms the null space of the ***S_I_***-matrix.

**Influence of biomass composition on growth yield:**

The influence of biomass composition on the predicted growth yield was assessed by the following procedure: a fraction of each compound in the biomass equation was varied both up and down by 20% and in each case, FBA was performed with the modified biomass equation set as the objective. The obtained values were compared with each other.

**Dependency of growth yield on Growth-Associate Maintenance (GAM) and Non-Growth-Associated Maintenance (NGAM):**

GAM and NGAM are two parameters that are related to non-metabolic energetic expenditures of the living cell [1] that, apart from the biomass composition and network structure, define maximal *in silico* growth yield. GAM is the energy expenditure necessary for non-metabolic activities accompanying the synthesis of a unit of biomass. NGAM is the energy expenditure that is used by the cell to maintain its life functions without growing. Their values are estimated from experimentally measured maximal yield and maintenance, respectively. The relationship between these values and the growth yield has the following form:

$$Y=\frac{Y_{atp}}{GAM+\frac{Y_{atp}}{Y_{\max}}}-\frac{NGAM}{\left(GAM+\frac{Y_{atp}}{Y_{\max}}\right)\cdot C_{\sup}}$$

where:

*Y_max_* – maximal *in silico* growth yield on carbon source (without GAM and NGAM) in *g_DW_·mmol^-1^*

*Y_atp_* – maximal *in silico* ATP yield on carbon source *mmol · mmol^-1^*

*C_sup_* – rate of supply of carbon source *mmol · g_DW_ · h^-1^*

It should be noted that the value of ATP yield (Y_atp_) depends on the stoichiometry of the oxidative chain – the P:O ratio (number of ATP molecules synthesized per mole of oxygen atoms reduced). This ratio was assumed to be equivalent to that used in the *E. coli* metabolic model (1.33) [2], as information regarding the stoichiometry of the oxidative chain in *P. putida* is not available.

**Comparison of FVA analyses with ^13^C flux measurement data:**

Fluxes estimated for *P. putida* central metabolism by the means of ^13^C measurements [3] were compared with *in silico* FVA flux predictions in iJP815. The ^13^C flux predictions derive from fitting of reaction fluxes to a metabolic network model, all the while accounting for flux ratios computed from the distribution of amino acid isotopomers [4]. The metabolic network used for these computations is a section of the metabolic network of an organism and the reactions building it should be a subset of the metabolic reconstruction. However, we could find no genetic evidence for the presence in *P. putida* of one reaction (phosphoenolpyruvate carboxykinase) that was in the ^13^C flux model, and one reaction (pyruvate carboxylase) that was irreversible in the ^13^C flux model was allowed to be reversible in iJP815. Additionally, the authors of the ^13^C work assume explicitly the absence of two reactions (phosphoenolpyruvate carboxylase and oxaloacetate carboxy-lyase) that are present in iJP815. We investigated these differences and according to available data, our version of the metabolic network structure is correct.

The uptake of glucose can proceed in *P.*putida on three different routes [5]. As the ^13^C measurements are not able to distinguish between these routes, it was assumed in the original work that the glucose is taken up directly. The same assumption was made in the optimal FVA analysis. In the suboptimal FVA analysis, all three routes of uptake were allowed.

**Influence of biomass composition on the outcome of FBA/FVA simulations:**

FVA was performed for each biomass equation created in the section “Influence of biomass composition on growth yield”. The minimal and maximal flux values from these simulations were used to estimate the possible range of flux ratios measured in ^13^C flux experiments [4]. For each flux ratio and biomass equation the lowest and the highest possible value of the *in silico* flux ratio was estimated, while keeping the *in silico* growth yield equal to that assumed in FVA computations. Subsequently, for every flux ratio, the mean and standard deviation of the minimal and maximal values across all the biomass compositions were computed. Afterwards suboptimal FVA was performed and the same computations were repeated for its results.

**Flux Coupling Finder (FCF):**

FCF is a method based on linear programming that assesses the correlative relationships among flux values in a network [6]. FCF identifies three types of relationships between reactions: directional coupling (*v_1_*→*v_2_*), whereby a non zero flux over one reaction (*v_1_*) implies a non-zero flux over the other (*v_2_*) but the converse is not necessarily true; partial coupling (*v_1_*↔*v_2_*), whereby a non-zero flux on either reaction implies a non-zero flux on the other, but the flux ratio can take any value from a certain interval; and full coupling (*v_1_*⇔*v_2_*), whereby a defined flux on one reaction implies a defined (but not necessarily equal) flux on the other. In the case of full coupling, a zero flux for one reaction always implies a zero flux for the other. It is worth noting that while all three types of coupling are transitive (i.e., *v_1_*→*v_2_* and *v_2_*→*v_3_* implies *v_1_*→*v_3_*), only partial coupling and full coupling are commutative (i.e., *v_1_*⇔*v_2_* implies *v_2_*⇔*v_1_*). Aside from its value in measuring metabolic network flexibility, FCF can aid in choosing mutational strategy, e.g., in choosing the easiest gene to manipulate out of a set coding for reactions in a fully coupled set. It can also narrow down the space of possible fluxes predicted by FVA, since reactions belonging to one fully coupled set always behave in a concerted way. This means that if the flux of one reaction from a fully coupled set is set at some value, the fluxes of remaining reactions in the set become then fixed. Sets of fully coupled reaction correspond to “correlated subsets” [7] that can be identified via Extreme Pathway analysis [8]. FCF can be described by the following pseudo-code:

1. Identify blocked reactions (e. g. by running FBA) and exclude them from further analysis,
2. Set *InCoupledSet*=∅
3. For *i* = {*1* to *N*-*1*} (where *N* is the number of reactions to be analyzed) do:
   1. If *v_i_∈InCoupledSet* then start the next iteration.
   2. For j = {*i*+*1* to *N*} do:
      1. set flux over the *v_j_* to 1;
      2. using FBA find minimal (*v_min_*) and maximal (*v_max_*) value of *v_i_*;
      3. if *v_min_*=*0* and v_max_ is unbounded then reactions are uncoupled;
      4. else if *v_min_*=*0* and *v_max_*=*c*>*0* then *v_i_*→*v_j_*;
      5. else if *v_min_=c>0* and *v_max_* is unbounded then *v_j_*→*v_i_*;
      6. else if *v_min_=c_1_>0* and *v_max_=c_2_>0* then:
         1. put reaction *v_j_* to the set of reactions coupled with *v_i_*;
         2. put reaction into *InCoupledSet;*
         3. if *c_2_-c_1_>0* then *v_1_*↔*v_2_*;
         4. if *c_2_-c_1_=0* then *v_1_*⇔*v_2_*;

**OptKnock – mathematical formulation:**

OptKnock extends from the FBA optimization framework by utilizing the duality property in linear programming [9]. Given the set of fluxes ***v***, the stoichiometric matrix ***S***, the vector of limits on particular fluxes ***l*** and the vector of contribution of particular fluxes in the objective ***c****_p_*, the original FBA problem can be written as:

maximize: $c_{p}^{T}\cdot v$ subject to:

$$S\cdot v=\mathbf{0}$$

$$L\cdot v\leq l$$

where ***L*** is the binary (and usually diagonal) matrix showing to which reaction a particular limit apply.

The matrices ***S*** and ***L*** have the same number of columns and can be put together to form a matrix of the linear problem:

$\left[\begin{matrix} S \\ L \end{matrix}\right]=A=\left[\begin{matrix} R & \end{matrix}I\right]$
where ***R*** and ***I*** are the submatrices pertaining the reversible and irreversible reactions, respectively.

Then the dual problem can be written:

minimize: subject to:

$$\lambda^{T}\cdot R=c_{p,rev}^{T}$$

$$\lambda^{T}\cdot I=c_{p,irrev}^{T}$$

$$\lambda_{stoch}\in\left[-\infty,\infty\right]$$

$$\lambda_{ineq}\geq0$$

where:

***c****_d_* – is the dual objective and $c_{d}^{T}=\left[c_{d,eq},c_{d,ineq}\right]^{T}$, where ***c****_d,eq_=****0*** and **c**_d,ineq_=***l***

***λ*** – are the dual variables, where $\lambda^{T}=\left[\lambda_{stoch},\lambda_{ineq}\right]^{T}$, and ***λ****_stoch_* and ***λ****_ineq_* – are the dual variables related to the primal stoichiometric (metabolites) and the inequality (flux limiting) constraints, respectively.

***c****_p,rev_* and ***c****_p,irrev_* – are vectors indicating the participation of the reversible and irreversible reactions in the primal objective, respectively, and $c_{p}^{T}=\left[c_{p,rev},c_{p,irrev}\right]^{T}$

The OptKnock problem can be than stated:
maximize: $c_{O}^{T}\cdot v$ (outer objective)
subject to: $c_{p}^{T}\cdot v=\lambda^{T}\cdot c_{d}$ (existence of inner objective)

$$S\cdot v=\mathbf{0}\equiv c_{d,eq}$$

$$L\cdot v\leq c_{d,ineq}$$

$$\lambda^{T}\cdot R=c_{p,rev}^{T}$$

$$\lambda^{T}\cdot I=c_{p,irrev}^{T}$$

$$\lambda_{stoch}\in\left[-\infty,\infty\right]$$

$$\lambda_{ineq}\geq0$$

$$y_{i}\cdot v_{i}^{\min}\leq v_{i}\leq y_{i}\cdot v_{i}^{\max}$$

$$\forall i\in B$$

$$\lambda_{ineq,i}^{\max}+\lambda_{ineq,i}^{\min}\leq\lambda_{i}^{\max}\cdot\left(1-y_{i}\right)$$

$\sum_{i} \left(1-y_{i}\right)\leq K$
where:

***c****_O_* – is the OptKnock objective

***y****_i_* – is the binary variable defining the activity of particular reaction

***B*** – is the set of reactions that can be deactivated

*K* – is the limit of the number of the reactions that can be inactivated

***v****_i_^min^*, ***v****_i_^max^*, ***λ****_i_^max^* – are limits on the corresponding variables

**REFERENCES**

1. Varma A, Palsson BO (1994) Stoichiometric Flux Balance Models Quantitatively Predict Growth and Metabolic by-Product Secretion in Wild-Type *Escherichia coli* W3110. Applied and Environmental Microbiology 60: 3724-3731.

2. Reed JL, Vo TD, Schilling CH, Palsson BO (2003) An expanded genome-scale model of *Escherichia coli* K-12 (iJR904 GSM/GPR). Genome Biology 4: R54.

3. Fuhrer T, Fischer E, Sauer U (2005) Experimental identification and quantification of glucose metabolism in seven bacterial species. Journal of Bacteriology 187: 1581-1590.

4. Fischer E, Zamboni N, Sauer U (2004) High-throughput metabolic flux analysis based on gas chromatography-mass spectrometry derived C-13 constraints. Analytical Biochemistry 325: 308-316.

5. Lessie TG, Phibbs PV (1984) Alternative Pathways of Carbohydrate Utilization in Pseudomonads. Annual Review of Microbiology 38: 359-387.

6. Burgard AP, Nikolaev EV, Schilling CH, Maranas CD (2004) Flux coupling analysis of genome-scale metabolic network reconstructions. Genome Research 14: 301-312.

7. Papin JA, Price ND, Palsson BO (2002) Extreme pathway lengths and reaction participation in genome-scale metabolic networks. Genome Research 12: 1889-1900.

8. Schilling CH, Letscher D, Palsson BO (2000) Theory for the systemic definition of metabolic pathways and their use in interpreting metabolic function from? A pathway-oriented perspective. Journal of Theoretical Biology 203: 229-248.

9. Burgard AP, Pharkya P, Maranas CD (2003) OptKnock: A bilevel programming framework for identifying gene knockout strategies for microbial strain optimization. Biotechnology and Bioengineering 84: 647-657.
