# Supplementary material for: Genome-Scale Reconstruction and Analysis of the Pseudomonas putida KT2440 Metabolic Network Facilitates Applications in Biotechnology
Source: PLoS Comput Biol. 2008 Oct 31;4(10):e1000210. doi: 10.1371/journal.pcbi.1000210 (PMC2563689; doi:10.1371/journal.pcbi.1000210)
Supplement: Text S2 — Supplementary results (0.05 MB DOC) [file pcbi.1000210.s013.doc]

**SUPPLEMENTARY RESULTS**

**Comparison of FVA analyses with ^13^C flux measurement data**

We modified iJP815 to comply with assumptions made in the metabolic network used in the ^13^C study [1], and performed FVA in the same way as for the unmodified network of iJP815. The results are depicted in the Supplementary Figure 3. The differences in predictions of attainable flux values, when compared with unmodified network, pertain mainly to reactions that were directly affected by the network modification. The only important difference is partial inactivation of the glyoxylate shunt in optimal FVA predictions, showing that the equality of yield between glyoxylate shunt and TCA cycle depends on one of the removed or modified reactions. In suboptimal FVA the glyoxylate shunt could be fully activated again, showing that the network modification did not lead to its full inactivation but rather slightly decreased it energetic yield.

**Influence of the biomass composition on the distribution of internal fluxes.**

We evaluated the influence of biomass composition on central metabolic fluxes. In this analysis we used flux ratios, which are the direct outcomes of ^13^C experiments [2], to compare *in silico* ratio values directly with experimental measurements. For each flux ratio the mean and standard deviation of both minimal and maximal possible value were estimated. The values were then plotted against each other and against experimentally defined flux ratios (see Supplementary Figure 4A). The same procedure was followed for suboptimal FVA (Supplementary Figure 4B). The spread between the means is a function of the network structure whereas the standard deviations show the influence of the biomass composition on the flux ratio itself. As these deviations were small for all flux ratios, this suggests that variations in biomass composition do not have significant impact on central metabolic fluxes. The experimental flux ratio values either fell between the minimal and maximal values or did not differ much from them. In the two cases where the experimental values did not fall between minimum and maximum the difference between the border of the *in silico* interval and the experimental value was very small. The upper and lower borders of the interval for the “Serine from glycolysis” ratio both fell below zero (as it is a ratio they both should be between zero and one). This suggested that in the reconstruction there existed one or more reactions contributing to production or consumption of L-serine that were not included in the computed ratio. Computing flux ratio ranges from sub-optimal FVA yielded far broader intervals in most cases, although the standard deviations were still small. On the other hand, more values fell out of the zero-one interval, suggesting again that more reactions existed producing the compounds related to the flux ratio than the ones accounted for in computing the ratio. The broad spread between the corresponding minima and maxima is an additional indication that the prediction of internal fluxes with FBA may lead to false results.

**Analysis of flexibility of the flux over particular reactions**

Aside from the ability of FVA to assess redundancy of a metabolic network as described in the main text, it can also shed light on network structure as a whole. Therefore, we performed a more in-depth analysis of the network structure of *P. putida* metabolism utilizing FVA. In the outcome of FVA one can observe five different behaviors, according to which we divided the reactions into five groups: (1) reactions with no flux, (2) reactions with non-variable non-zero flux, (3) reactions in which flux can vary, but cannot be zero, (4) reactions that can have a zero flux, and (5) reactions that can operate reversibly, i.e., that can have positive, negative, or zero flux (only reversible reactions may belong to this group). FVA simulations were performed in both optimal and suboptimal versions (Supplementary Figure 5) for two carbon sources, glucose and acetate, which are glycolytic and gluconeogenic, respectively. These two types of carbon sources have different mode of utilization of certain parts of central metabolism (Glycolysis, Pentose Phosphate Pathway, Entner-Doudoroff Pathway), allowing us to identify differences in flexibility between these two modes.

Analysis of the sizes of the groups indicated that zero-flux reactions constitute in all cases the biggest group, reaching 47 percent of reactions in the network. This is the consequence of the high number of the “unconditionally blocked” reactions (289, as estimated before). A significant difference can be observed for this group between optimal and suboptimal analyses. This is due to the fact that the groups created from the results of optimal analyses contain, apart from “unconditionally blocked” reactions, reactions blocked due to the limits imposed on the metabolic network (about 60 reactions) and reactions with negative influence on the objective (growth yield; about 40 reactions). The groups created from suboptimal analyses contain only the former subgroup. No reactions with fixed non-zero flux appear in the suboptimal analyses, which is a natural consequence of allowed variability of the growth yield in this suboptimal case. This group contains the reactions belonging to pathways which do not have an alternative with equal efficiency. The next group (reactions in which flux can vary, but cannot be zero), when it is created from an optimal analysis, is composed of reactions that take part in multiple processes, part of which, but not all, can be substituted by some other that operate with the same efficiency. When it is created from sub-optimal analysis it contains part of the reactions that belong to groups 2 and 3 in optimal analysis. The fourth and fifth groups (reactions that can have a zero flux and reactions that can operate reversibly, respectively) contain reactions that can be substituted by some others, with equal or similar efficiency.

Comparison of acetate and glucose simulations shows that the conditions differ rather insignificantly. Growth on glucose allows for higher flexibility of the metabolic network, as the sizes of fourth and fifth groups are greater in these conditions, while the remaining groups are smaller.

We also assessed how the flexibility is distributed among pathways. In Supplementary Table 3, we rank-ordered pathways in iJP815 by their fraction of reactions whose fluxes are variable during FVA simulations (i.e., reactions belonging to groups 4 or 5). Reactions were assigned to pathways by mapping iJP815 to KEGG reactions and assigning KEGG pathways identifications to all model reactions for which a KEGG reaction could be identified. The analysis was performed only for KEGG pathways that shared at least ten reactions with iJP815. There exists no obvious pattern regarding the variability of reactions in different pathways, although it appears that biosynthetic pathways show higher rigidity.

**Identification of coupled reactions using Flux Coupling Finder (FCF).**

To assess the presence of coupled reactions iJP815, we utilized the flux coupling finder (FCF) approach. FCF is similar to extreme pathway (EP) and elementary mode (EM) approaches in that it lends insight into the functional relationships between reactions in a network, but its computational requirements are much lower and therefore it is applicable to large models such as iJP815.

As flux couplings in the metabolic network depend on *in silico* availability of external metabolites, FCF analysis was performed for minimal medium conditions with both glucose and acetate as sole carbon sources (Supplementary Figure 6A and 6B). FCF was performed both treating biomass constituents as independent sinks and treating biomass as a complete reaction equal to the properly weighted sum of all biomass components (as described previously). Similar numbers of reactions belonged to coupled reaction sets (CoSets) for glucose and acetate growth (462 and 465 on glucose and acetate respectively, 422 and 425 when biomass constituents could be sinked independently). These numbers correspond to about 90% of non-blocked reactions. Most CoSets consist of unbroken consecutive chains of reactions, and since the *P. putida* metabolic network shows a high degree of interconnectedness, most CoSets contain less than 5 reactions, The mean size of CoSets varied between 3 and 5, being more than one unit lower in the computations wherein biomass was not fixed (3.58 and 3.73 for glucose and acetate conditions, respectively) than in the counterpart with fixed biomass (4.86 and 5.17 for glucose and acetate conditions, respectively). This average is slightly lower under glucose versus acetate conditions, which also indicates that glucose growth conditions exhibit lower network rigidity than acetate growth conditions. The biggest CoSet, containing 185 reactions under both acetate and glucose growth with fixed biomass composition, consisted of reactions coupled to the biomass reaction. It is worth noting that the reactions coupled in any way (fully, partially or directionally) to biomass compose the set of reactions essential for growth as identified earlier by other means.

We assessed whether the full couplings are able to limit the ranges of flux ratio values used to compare with ^13^C experiments by coupling appropriate FVA ranges together. However, there exist no couplings among reactions involved in computation of these ratios (data not shown).

**REFERENCES**

1. Fuhrer T, Fischer E, Sauer U (2005) Experimental identification and quantification of glucose metabolism in seven bacterial species. Journal of Bacteriology 187: 1581-1590.

2. Fischer E, Zamboni N, Sauer U (2004) High-throughput metabolic flux analysis based on gas chromatography-mass spectrometry derived C-13 constraints. Analytical Biochemistry 325: 308-316.
