# Supplementary material for: Genome-Scale Reconstruction and Analysis of the Pseudomonas putida KT2440 Metabolic Network Facilitates Applications in Biotechnology
Source: PLoS Comput Biol. 2008 Oct 31;4(10):e1000210. doi: 10.1371/journal.pcbi.1000210 (PMC2563689; doi:10.1371/journal.pcbi.1000210)
Supplement: Text S3 — Publications that contributed to the iJP815 reconstruction process (0.02 MB DOC) [file pcbi.1000210.s014.doc]

1. Alaminos M, Ramos JL (2001) The methionine biosynthetic pathway from homoserine in Pseudomonas putida involves the metW, metX, metZ, metH and metE gene products. Archives of Microbiology 176: 151-154.
2. Arias-Barrau E, Olivera ER, Luengo JM, Fernandez C, Galan B, et al. (2004) The homogentisate pathway: A central catabolic pathway involved in the degradation of L-phenylalanine, L-tyrosine, and 3-hydroxyphenylacetate in Pseudomonas putida. Journal of Bacteriology 186: 5062-5077.
3. Chang YF, Adams E (1974) D-Lysine Catabolic Pathway in Pseudomonas putida - Interrelations with L-Lysine Catabolism. Journal of Bacteriology 117: 753-764.
4. del Castillo T, Ramos JL, Rodriguez-Herva JJ, Fuhrer T, Sauer U, et al. (2007) Convergent peripheral pathways catalyze initial glucose catabolism in Pseudomonas putida: Genomic and flux analysis. Journal of Bacteriology 189: 5142-5152.
5. Duetz WA, Wind B, van Andel JG, Barnes MR, Williams PA, et al. (1998) Biodegradation kinetics of toluene, m-xylene, p-xylene and their intermediates through the upper TOL pathway in Pseudomonas putida (pWW0). Microbiology-Sgm 144: 1669-1675.
6. Gibson DT, Koch JR, Kallio RE (1968) Oxidative Degradation of Aromatic Hydrocarbons by Microorganisms .I. Enzymatic Formation of Catechol from Benzene. Biochemistry 7: 2653-&.
7. Harder W, Quayle JR (1971) Aspects of Glycine and Serine Biosynthesis during Growth of Pseudomonas Am1 on C1 Compounds. Biochemical Journal 121: 763-&.
8. Harder W, Quayle JR (1971) Biosynthesis of Serine and Glycine in Pseudomonas Am1 with Special Reference to Growth on Carbon Sources Other Than C1 Compounds. Biochemical Journal 121: 753-&.
9. Heptinst.J, Quayle JR (1970) Pathways Leading to and from Serine during Growth of Pseudomonas Am1 on C1 Compounds or Succinate. Biochemical Journal 117: 563-&.
10. Ito K, Takahashi M, Yoshimoto T, Tsuru D (1994) Cloning and High-Level Expression of the Glutathione-Independent Formaldehyde Dehydrogenase Gene from Pseudomonas putida. Journal of Bacteriology 176: 2483-2491.
11. Jeffrey WH, Cuskey SM, Chapman PJ, Resnick S, Olsen RH (1992) Characterization of Pseudomonas putida Mutants Unable to Catabolize Benzoate - Cloning and Characterization of Pseudomonas Genes Involved in Benzoate Catabolism and Isolation of a Chromosomal DNA Fragment Able to Substitute for Xyls in Activation of the Tol Lower-Pathway Promoter. Journal of Bacteriology 174: 4986-4996.
12. Jimenez JI, Minambres B, Garcia JL, Diaz E (2002) Genomic analysis of the aromatic catabolic pathways from Pseudomonas putida KT2440. Environmental Microbiology 4: 824-841.
13. Kato N, Kobayashi H, Shimao M, Sakazawa C (1984) Properties of Formaldehyde Dismutation Catalyzing Enzyme of Pseudomonas putida F61. Agricultural and Biological Chemistry 48: 2017-2023.
14. Kato N, Yamagami T, Shimao M, Sakazawa C (1986) Formaldehyde Dismutase, a Novel Nad-Binding Oxidoreductase from Pseudomonas putida F61. European Journal of Biochemistry 156: 59-64.
15. Kim YH, Cho K, Yun SH, Kim JY, Kwon KH, et al. (2006) Analysis of aromatic catabolic pathways in Pseudomonas putida KT 2440 using a combined proteomic approach: 2-DE/MS and cleavable isotope-coded affinity tag analysis. Proteomics 6: 1301-1318.
16. Kopchick JJ, Hartline RA (1979) Alpha-Hydroxyglutarate as an Intermediate in the Catabolism of Alpha-Aminoadipate by Pseudomonas putida. Journal of Biological Chemistry 254: 3259-3263.
17. Kumar A, Kumar S, Kumar S (2005) Biodegradation kinetics of phenol and catechol using Pseudomonas putida MTCC 1194. Biochemical Engineering Journal 22: 151-159.
18. Large PJ, Quayle JR (1963) Microbial Growth on C1 Compounds .5. Enzyme Activities in Extracts of Pseudomonas Am1. Biochemical Journal 87: 386-&.
19. Leidigh BJ, Wheelis ML (1973) Genetic Control of Histidine Dissimilatory Pathway in Pseudomonas putida. Molecular & General Genetics 120: 201-210.
20. Luengo JM, Garcia JL, Olivera ER (2001) The phenylacetyl-CoA catabolon: a complex catabolic unit with broad biotechnological applications. Molecular Microbiology 39: 1434-1442.
21. Martin RR, Marshall VD, Sokatch JR, Unger L (1973) Common Enzymes of Branched-Chain Amino-Acid Catabolism in Pseudomonas putida. Journal of Bacteriology 115: 198-204.
22. Massey LK, Conrad RS, Sokatch JR (1974) Regulation of Leucine Catabolism in Pseudomonas putida. Journal of Bacteriology 118: 112-120.
23. Molin G, Nilsson I (1985) Degradation of Phenol by Pseudomonas putida Atcc-11172 in Continuous Culture at Different Ratios of Biofilm Surface to Culture Volume. Applied and Environmental Microbiology 50: 946-950.
24. Morris JG (1969) Utilization of L-Threonine by a Pseudomonad - a Catabolic Role for L-Threonine Aldolase. Biochemical Journal 115: 603-&.
25. Muramatsu H, Mihara H, Kakutani R, Yasuda M, Ueda M, et al. (2005) The putative malate/lactate dehydrogenase from Pseudomonas putida is an NADPH-dependent Delta(1)-piperideine-2-carboxylate/Delta(1)-pyrroline-2-carboxylate reductase involved in the catabolism of D-lysine and D-proline. Journal of Biological Chemistry 280: 5329-5335.
26. Nogales J, Macchi R, Franchi F, Barzaghi D, Fernandez C, et al. (2007) Characterization of the last step of the aerobic phenylacetic acid degradation pathway. Microbiology-Sgm 153: 357-365.
27. Otenio MH, da Silva MTL, Marques MLO, Roseiro JC, Bidoia ED (2005) Benzene, toluene and xylene biodegradation by Pseudomonas putida CCMI 852. Brazilian Journal of Microbiology 36: 258-261.
28. Payton CW, Chang YF (1982) Delta-1-Piperideine-2-Carboxylate Reductase of Pseudomonas putida. Journal of Bacteriology 149: 864-871.
29. Perfetti R, Titus J, Hartline RA, Campbell RJ (1972) Catabolism of Pipecolate to Glutamate in Pseudomonas putida. Journal of Biological Chemistry 247: 4089-&.
30. Petruschka L, Adolf K, Burchhardt G, Dernedde J, Jurgensen J, et al. (2002) Analysis of the zwf-pgl-eda-operon in Pseudomonas putida strains H and KT2440. Fems Microbiology Letters 215: 89-95.
31. Revelles O, Espinosa-Urgel M, Fuhrer T, Sauer U, Ramos JL (2005) Multiple and interconnected pathways for L-lysine catabolism in Pseudomonas putida KT2440. Journal of Bacteriology 187: 7500-7510.
32. Revelles O, Espinosa-Urgel M, Molin S, Ramos JL (2004) The davDT operon of Pseudomonas putida, involved in lysine catabolism, is induced in response to the pathway intermediate delta-aminovaleric acid. Journal of Bacteriology 186: 3439-3446.
33. Revelles O, Wittich RM, Ramos JL (2007) Identification of the initial steps in D-lysine catabolism in Pseudomonas putida. Journal of Bacteriology 189: 2787-2792.
34. Riis V, Miethe D, Babel W (2003) Formate-stimulated oxidation of methanol by Pseudomonas putida 9816. Bioscience Biotechnology and Biochemistry 67: 684-690.
35. Roberts CM, Conrad RS, Sokatch JR (1978) Role of Enoyl-Coa Hydratase in Metabolism of Isoleucine by Pseudomonas putida. Archives of Microbiology 117: 99-108.
36. Sawyer MH, Baumann P, Baumann L, Berman SM, Canovas JL, et al. (1977) Pathways of D-Fructose Catabolism in Species of Pseudomonas. Archives of Microbiology 112: 49-55.
37. Shimizu S, Ohshiro T, Shiozaki S, Yamada H (1986) Production of S-Adenosyl-L-Homocysteine by Bacterial-Cells with a High Content of S-Adenosylhomocysteine Hydrolase - Utilization of a Racemic-Mixture of Homocysteine as the Substrate. Journal of Biotechnology 4: 91-100.
38. Sykes PJ, Menard J, Mccully V, Sokatch JR (1985) Conjugative Mapping of Pyruvate, 2-Ketoglutarate, and Branched-Chain Keto Acid Dehydrogenase Genes in Pseudomonas putida Mutants. Journal of Bacteriology 162: 203-208.
39. Toyama H, Fujii A, Matsushita K, Shinagawa E, Ameyama M, et al. (1995) 3 Distinct Quinoprotein Alcohol Dehydrogenases Are Expressed When Pseudomonas putida Is Grown on Different Alcohols. Journal of Bacteriology 177: 2442-2450.
40. Tricot C, Stalon V, Legrain C (1991) Isolation and Characterization of Pseudomonas putida Mutants Affected in Arginine, Ornithine and Citrulline Catabolism - Function of the Arginine Oxidase and Arginine Succinyltransferase Pathways. Journal of General Microbiology 137: 2911-2918.
41. Vilchez S, Manzanera M, Ramos JL (2000) Control of expression of divergent Pseudomonas putida put promoters for proline catabolism. Applied and Environmental Microbiology 66: 5221-5225.
42. Vilchez S, Molina L, Ramos C, Ramos JL (2000) Proline catabolism by Pseudomonas putida: Cloning, characterization, and expression of the put genes in the presence of root exudates. Journal of Bacteriology 182: 91-99.
43. Vilchez S, Molina L, Ramos C, Ramos JL (2000) Proline catabolism by Pseudomonas putida: Cloning, characterization, and expression of the put genes in the presence of root exudates. Journal of Bacteriology 182: 91-99.
44. Wheelis ML, Stanier RY (1970) Genetic Control of Dissimilatory Pathways in Pseudomonas putida. Genetics 66: 245-&.
45. Yanase H, Noda H, Aoki K, Kita K, Kato N (1995) Cloning, Sequence-Analysis, and Expression of the Gene Encoding Formaldehyde Dismutase from Pseudomonas putida F61. Bioscience Biotechnology and Biochemistry 59: 197-202.
